# Food, Quality of Life and Mental Health: A Cross-Sectional Study with Federal Education Workers

**DOI:** 10.3390/nu17152519

**Published:** 2025-07-31

**Authors:** José Igor Ferreira Santos Jesus, Manuel Monfort-Pañego, Gabriel Victor Alves Santos, Yasmin Carla Monteiro, Suelen Marçal Nogueira, Priscilla Rayanne e Silva, Matias Noll

**Affiliations:** 1 Postgraduate Program in Nutrition and Health (PPGNUT), Faculty of Nutrition, Federal University of Goiás (UFG), Goiânia 74605-080, GO, Brazil; 2 Physical Education Teacher Education Department, Teacher Education Faculty, University of Valencia, 46010 Valencia, Spain; manuel.monfort@uv.es; 3 Research Department, Goiano Federal Institute, Campus Ceres, Ceres 76300-000, GO, Brazil; gabriel.victor2@estudante.ifgoiano.edu.br (G.V.A.S.); yasmin.monteiro@estudante.ifgoiano.edu.br (Y.C.M.); suelen.nogueira@ifgoiano.edu.br (S.M.N.); priscilla.silva@ifgoiano.edu.br (P.R.e.S.); 4 Physiotherapy Department, Universidade Evangélica de Goiás, Campus Ceres, Ceres 76300-000, GO, Brazil

**Keywords:** lifestyle, mental health, occupational health, quality of life, ultra-processed foods

## Abstract

**Background**: The consumption of ultra-processed foods (UPFs) represents an important public health challenge, especially among education workers, whose intense routine can negatively impact eating habits. This study aimed to analyze the factors associated with the regular consumption of UPF among employees of the Federal Network of Professional, Scientific and Technological Education (RFEPCT) in Brazil. **Methods**: This was a cross-sectional study, with a quantitative approach, carried out with 1563 education workers. Validated instruments on eating habits (PeNSE), mental health (DASS-21) and quality of life (WHOQOL-bref) were used. The regular consumption of UPF was defined as intake on ≥5 days in the last seven days. The association between the regular consumption of UPF and sociodemographic, occupational, behavioral, mental health and quality of life variables was assessed by Poisson regression with robust variance, generating adjusted prevalence ratios (PRadj) and respective 95% confidence intervals. **Results**: The regular consumption of UPF was associated mainly with female gender, a lower age group, Southeast and Midwest regions, dissatisfaction with sleep and the body, physical inactivity and poor sleep quality. In addition, the findings suggested a significant relationship between the worst stress scores and soft drinks (PRadj: 2.11; CI: 1.43–3.13), anxiety and soft drinks (PRadj: 1.83; CI: 1.24–2.70) and depression and industrialized/ultra-processed salty foods (PRadj: 2.43; CI: 1.82–3.26). The same was observed in the scores for the worst perception of quality of life, where there was a prevalence of up to 2.32 in the psychological domain and the consumption of industrialized/ultra-processed salty foods. **Conclusions**: The findings indicate that multiple interrelated factors—individual, psychosocial and occupational—are associated with the consumption of UPF among education workers. These results reinforce the importance of institutional policies that integrate actions to promote dietary health, mental health care and improved working conditions in the education sector.

## 1. Introduction

The increased consumption of ultra-processed foods (UPFs) is a global concern due to its association with various health problems [1,2], such as obesity [3], diabetes, cardiovascular diseases [4] and psychological disorders [5,6]. In addition, studies show that the higher consumption of UPF is inversely associated with protein, fiber, fruit and vegetable intake [7,8]. This scenario is relevant among education workers, whose routine is often marked by high emotional demand, intense workload and challenges related to reconciling professional and personal life [9,10].

UPF consumption has been widely associated with negative outcomes related to quality of life and mental health [11]. Studies indicate that diets rich in UPF, often poor in essential nutrients, can contribute to increased symptoms of anxiety, depression and stress [12,13]. This dietary pattern, in turn, can directly impact the perception of physical [14], psychological [15] and social well-being [16]. Psychologically, negative emotional states can lead to the consumption of comfort foods and less healthy food choices, creating a two-way cycle between diet and mental health [17,18]. In addition, the low nutritional density and high levels of additives present in these foods can intensify inflammatory processes [19,20], affecting both mental health and quality of life [21].

The relationship between diet and mental health has been explained by different biological and psychological mechanisms [22]. These include the modulation of the gut–brain axis, systemic inflammatory processes, changes in the gut microbiota, nutritional deficiencies (especially in B vitamins, omega-3, magnesium and zinc) and the impact of the high glycemic index of UPF on mood regulation [12,17,18,23,24,25]. Observational studies and systematic reviews have shown that UPF-based diets are associated with a higher prevalence of depressive and anxiety symptoms, while healthy eating patterns, such as the Mediterranean diet, have been shown to have a protective effect against these same outcomes [26,27,28,29]. For example, the study by Dopelt K., Houminer-Klepar N. (2024) identified significant associations between greater adherence to the Mediterranean diet and lower levels of anxiety and depression in Israeli adults, suggesting an antagonistic effect of UPF consumption [30].

In addition to nutritional impacts, the consumption of UPF also reflects structural issues, such as limited access to fresh foods [31,32], a lack of awareness of the harm caused by the regular consumption of these foods [33] and the influence of advertising on eating behavior [34]. Recent studies point out that these foods, because they are practical and accessible, often replace balanced meals [8], especially in contexts of long working hours [35] and school environments that do not always promote healthy eating practices [36,37].

Despite the growing body of evidence on the impacts of UPF consumption on health [38,39,40], there are still few studies exploring this issue specifically among education workers [41,42]. This professional group faces unique challenges, such as a high workload [43] and emotional demands [44], which can influence eating habits [45] and, consequently, quality of life [46] and mental health [44]. In this way, investigating the factors associated with regular consumption of these foods in the educational context makes it possible not only to identify patterns of health and nutrition among these professionals [47] but also to subsidize public policies and institutional strategies aimed at promoting health and well-being in the workplace [9].

Adopting a quality diet among educators is essential for promoting physical and mental health [48,49], especially in the face of the emotional, cognitive and social demands of everyday life [50,51]. A balanced diet helps maintain energy and emotional balance, prevent chronic diseases and improve mental health [52]. In this context, public policies aimed at educators’ health, such as intersectoral actions that integrate healthy eating, psychosocial support and adequate working conditions, play a strategic role in well-being [53]. Educators who are healthy and valued tend to have better teaching performance, positively impacting the comprehensive education of students [36,54]. Therefore, this study aims to analyze the factors associated with the consumption of UPF and their relationship with quality of life and mental health among education workers.

## 2. Materials and Methods

This is a cross-sectional epidemiological study with a quantitative approach. This study was carried out at the Brazilian Federal Network for Professional, Scientific and Technological Education (RFEPCT), between June and November 2022, based on the survey entitled “Quality of Life in Brazilian Education—QoLE-BRA” [55]. The participants were duly informed about the purpose of the study, the confidentiality of the information provided and the procedures required for data collection, in addition to all signing and receiving a copy of the Informed Consent Form (ICF). The research project was approved by the Research Ethics Committee of the Instituto Federal Goiano on 3 March 2022 (Protocol CAAE No 52353621.3.0000.0036).

### 2.1. Research Context

The Federal Network for Professional, Scientific and Technological Education (RFEPCT) was established by Law No. 11.892, of 29 December 2008, with the aim of integrating, expanding and consolidating the provision of professional and technological education in Brazil [56]. The RFEPCT is made up of public institutions linked to the Ministry of Education (MEC) and is characterized by its decentralized, multi-curricular activities focused on technical and higher education, applied research and extension [56,57].

Its institutional profile is marked by the provision of courses at all levels of education—from initial training (secondary education integrated with technical courses) to continuing education to doctorates—prioritizing regional development, social inclusion and the verticalization of education. The network promotes the articulation between education, work, science and technology, acting in an inseparable way between teaching, research and extension [58,59].

The composition of the RFEPCT includes 64 institutions, including 38 Federal Institutes of Education, Science and Technology (IFs), 2 Federal Technological Education Centers (CEFETs), 22 technical schools linked to universities and to Pedro II College, and 1 technological university (UTFPR). By 2024, the network had 685 campuses spread across all 27 Brazilian states, serving approximately 1.5 million students. With its capillarity and commitment to public, free and high-quality education, the RFEPCT is one of the main instruments for democratizing access to knowledge and promoting sustainable development in the country [60,61].

### 2.2. Population and Sample

The population of this study was made up of 83517 RFEPCT civil servants, according to the Nilo Peçanha Platform (PNP) for the year 2021 [60]. The PNP functions as a virtual space for the collection, validation and dissemination of official RFEPCT statistics, with the aim of gathering information on teachers, students, ATE and financial staff at the Federal Network units. All civil servants were invited to take part in this study, whether they were Administrative Technicians in Education (ATEs) or teachers. ATEs are education employees who work in technical and operational activities involving administrative, academic and management support [62]. All individuals whose questionnaires had been fully answered were included in this study and those who did not answer or who answered incompletely were excluded.

To calculate the population sample, a 95% confidence level and a 3% margin of error were adopted, resulting in a minimum number of respondents of 1054 and an increase of 20%, taking into account losses and errors in filling out the questionnaires, so a minimum of 1265 participants were required. The final sample consisted of 1563 participants from all five of Brazil’s major regions: the Midwest, Northeast, North, Southeast and South.

### 2.3. Data Collection

The survey was carried out using Google Forms and sent to the e-mails of teachers and ATEs, according to the contacts available on the institutional pages. A sociodemographic questionnaire was used, including data on gender, age, marital status, level of education, region of work, position and length of service. Questions about body perception and lifestyle habits were also part of the questionnaire, using the following questions: “How satisfied are you with your body?” and “How satisfied are you with your sleep?”, both of which had Likert-type answers, ranging from 1 (not at all) to 5 (completely). With regard to lifestyle habits, they were asked the following: “How many hours of sleep do you get per night?” (≤4 h, 5 h, 6 h, …, >10 h per night); “How many hours per day do you sit watching television?” (<1 h, 1 h, 2 h, …, >8 h per day); “Do you practice any physical exercise or sport regularly?” (yes, no); “How many days do you practice exercise/sport per week?” (no day, 1 day, …, ≥5 days per week and can’t answer/depends on the week).

In addition, the following validated questionnaires were applied: (1) the World Health Organization Quality of Life Brief Version (WHOQOL-bref), (2) the Depression, Anxiety and Stress Scale (DASS-21) and (3) the National School Health Survey (PeNSE).

(1) World Health Organization Quality of Life Brief Version—WHOQOL-bref [63]: Created by the World Health Organization (WHO), this is a comprehensive tool used to assess quality of life in different groups and cultural contexts. This instrument was developed and adapted with the aim of providing a detailed overview of how people perceive their own quality of life in various areas, taking into account factors that influence their well-being. It includes 26 items distributed between the following: the physical domain, the psychological domain, social relationships, the environmental domain and total score. The answers are given on a Likert-type scale, ranging from 1 (not at all) to 5 (completely), using four types of scales (depending on the content of the question): intensity, capacity, frequency and evaluation. The higher the score is, the better the individual’s perception of their quality of life is [63,64,65].
(2) Depression, Anxiety and Stress Scale—DASS-21 [66]: This is a widely used tool for assessing symptoms of depression, anxiety and stress. It has been validated in various cultures and populations, demonstrating good validity and reliability in different cultural contexts. The answers are given on a 4-point Likert scale, ranging from 0 (“strongly disagree”) to 3 (“strongly agree”) taking into account the state of mental health in the last week. These items are also organized into subscales, regarding depression, anxiety and stress, with 7 items for each subscale. The score for each subscale is equal to the sum of the seven corresponding questions. The sum scores are multiplied by 2 to correspond to the original scale score in the DASS-42 [67]. For the anxiety subgroup, the score ranges of ≤7, 8 to 9, 10 to 14, 15 to 19 and ≥20 imply normal, mild, moderate, severe and very severe, respectively. For the stress subgroup, scores ranging ≤14, from 15 to 18, from 19 to 25, from 26 to 33 and ≥34 indicate normal, mild, moderate, severe and very severe, respectively. For the depression subgroup, scores ranging ≤9, from 10 to 13, from 14 to 20, from 21 to 27 and ≥28 reflect normal, mild, moderate, severe and very severe. The lower the score is, the lower the levels of depression, anxiety and stress are [68,69,70,71]. For statistical analysis, the data was categorized as normal, moderate and high [72,73].
(3) National School Health Survey—PeNSE [74]: This survey has been carried out since 2009, in partnership with the Instituto Brasileiro de Geografia e Estatística (IBGE) and with the support of the Ministry of Education (MEC). The questionnaire covers the four common risk factors for chronic non-communicable diseases (smoking, sedentary lifestyle, inadequate diet and alcohol consumption). Data is collected on, for example, mental health, sexual and reproductive health, oral health, food consumption, body image and the use of cigarettes, alcohol and drugs, among others [75,76]. For this study, we used questions related to eating habits, where we considered (1) the consumption of sweets (candies, chocolates, chewing gum, chocolates, lollipops), (2) soft drinks, (3) industrialized/ultra-processed salty foods (hamburgers, ham, mortadella, salami, sausage, instant noodles, packaged snacks, salty cookies) and (4) fast food (snack bars, hot dog stands, pizzerias, among others) in the last 7 days. These variables were assessed using the following question: “In the last 7 days, on how many days did you eat/drink …?”. Eight answers were available: “I didn’t eat/drink in the last 7 days”, “I ate/drank on 1 day (2, …,6) of the last 7 days” and “I ate/drank on every day of the last 7 days”. The raw data was categorized for analysis. For eating habits, a period of 0–4 days a week was considered non-regular consumption and 5–7 days a week as regular consumption [77,78,79,80,81,82,83]. Thus, the categorization enabled a structured assessment of the regular consumption of these foods.

### 2.4. Data Analysis

The dependent variables related to the regular consumption of UPF were analyzed using descriptive statistics (absolute and percentage) and Wald’s chi-square association test (bivariate analysis). The following factors (blocks) were considered as independent variables: sociodemographic, work and training, body perception and lifestyle habits, mental health and quality of life. The Poisson regression model with robust variance (crude analysis) was used to analyze the independent variables. The measure of effect was the prevalence ratio (PR) with its respective 95% confidence intervals (CI) (α = 0.05). The crude PR was obtained by Poisson regression [84,85].

The independent variables were then adjusted (PRadj) in a logistic regression analysis using the Poisson regression model with robust variance (95% CI; α = 0.05), and the results were adjusted for sociodemographic variables (gender, age group, level of education, region, position and length of service). Based on the potential confounding factors, the adjustment variables were selected and supported by methodological and statistical studies [84,86,87]. All analyses were conducted using SPSS software version 26.0.

## 3. Results

The sociodemographic analysis showed that the education workers studied were predominantly female (57.3%), aged between 36 and 41 years (30.2%), married (65%), with a PhD (40.7%), from the Midwest region (32.2%), in the position of Education Administrative Technician (56.2%), with a length of service between 6 and 10 years (43.1%) and living in urban areas (95.1%) (Table 1). With regard to regular consumption (Table 2), 25.3% of the participants consumed sweets, 14.5% industrialized foods, 8.4% soft drinks and 2.5% fast food.

The results of the bivariate analysis are presented in Supplementary Table S1. The adjusted prevalence ratio analysis (PRadj) (Table 3) indicated that the regular consumption of sweets was associated (*p* < 0.05) with sociodemographic factors (gender, age group and region of the country), work and training (job title), body perception and lifestyle habits (body satisfaction, sleep quality, physical activity, weekly frequency of physical activity), mental health (depression) and quality of life (physical and psychological domains).

In relation to regular soft drink consumption, sociodemographic factors (age group and region of the country), body perception and lifestyle habits (body satisfaction, sleep quality, TV time, physical activity and weekly frequency of physical activity), mental health (stress, anxiety and depression) and quality of life (physical, psychological and total score domains) were associated (*p* < 0.05).

Sociodemographic factors (age group and region of the country), work and training (level of education and job title), body perception and lifestyle habits (body satisfaction, sleep quality, hours of sleep, physical activity, and weekly frequency of physical activity), mental health (stress, anxiety and depression) and quality of life (physical, psychological, social and environmental domains and total score) were associated with the outcome (*p* < 0.05).

Finally, the outcome of regular fast food consumption was associated (*p* < 0.05) with sociodemographic factors (marital status) and body perception and lifestyle habits (body satisfaction and physical activity).

## 4. Discussion

As far as we know, this is the first study to investigate factors related to the regular consumption of ultra-processed foods by RFEPCT civil servants. The results of this study indicate that the regular consumption of UPF among civil servants is associated with several factors. It was found that women, younger individuals, those living in the South and Southeast, those with a low quality of life and those with a higher level of stress, anxiety and depression were more likely to consume sweets, soft drinks and salty ultra-processed foods.

### 4.1. Sociodemographic Factors, Work and Training

Our findings indicate that women consumed more sweets than men. Although some studies have found different results [88,89,90], the results corroborate other previous studies, which indicate that women tend to have a greater preference for sweet foods [91,92,93,94]. Among the possible explanations, consumption may be associated with hormonal factors [95], emotional factors [96] and the heavy workload of women compared to that of men [97,98].

The consumption of UPF is increasing over the years in different age groups and, consequently, there is a reduction in the intake of healthy foods [99,100,101,102]. We observed in our study that the older age group tended to consume fewer UPF, especially sweets, soft drinks and salty industrialized/ultra-processed foods. This phenomenon may be related to changes in eating habits, greater concern for health and the adoption of healthier eating patterns throughout life [103,104,105].

In general, civil servants from the South, Southeast and Central–West regions consumed more sweets, soft drinks and salty industrialized/ultra-processed foods than those from the North–Northeast. These findings may reflect differences in culture and access to these products, as well as regional socioeconomic aspects [106,107] that influence the population’s eating patterns.

Single individuals consumed more fast food than married individuals, which can be attributed to a less structured lifestyle for preparing home-cooked meals and greater adherence to more practical and quicker eating habits [108,109]. Studies show that eating habits are influenced by living alone. This is due to factors such as social isolation and the lack of a support network [109,110], and eating together is an essential part of socialization [111,112].

In relation to education, individuals with a master’s degree had a higher consumption of industrialized/ultra-processed salty foods, while teachers consumed more sweets and industrialized/ultra-processed salty foods compared to ATEs. This may reflect the specific work and lifestyle patterns of these groups, including greater workload and occupational stress [15,113,114]. Teachers perform a role with great attention from the school community and are often subject to stressful factors, such as long working hours and reduced time for leisure and physical activity, which also directly affect sleep and rest, eating habits and quality of life [41,115].

### 4.2. Sleep Quality, Body Image and Lifestyle Habits

Individuals who were dissatisfied with their sleep had a higher consumption of UPF in most of the food categories analyzed. The impact of sleep quality on diet has been widely documented in the literature [116,117,118]. Sleep deprivation or dissatisfaction can lead to hormonal imbalances, such as an increase in ghrelin (hunger hormone) and a decrease in leptin (satiety hormone) [119], resulting in a greater desire for UPF [16]. In addition, tiredness and fatigue associated with poor sleep quality can lead to more practical and less healthy food choices, favoring the consumption of industrialized, energy-rich and easily consumed foods [120,121]. We also observed that individuals who slept less (≤6 h per night) consumed more industrialized/ultra-processed salty foods, which may be related to the influence of sleep on physiological mechanisms of hunger and satiety [16]. Sleep deprivation or insomnia can be predictive of the development of depression [122,123] and decreased alertness [124], as well as a higher risk of cardiovascular disease [125,126], obesity, hypertension, diabetes, stroke, frequent mental suffering and death [127].

A sedentary lifestyle or physical inactivity combined with the consumption of UPF is linked to chronic diseases and premature death [128]. On the other hand, physical activity can have a significant impact on reducing disease [129,130,131] and on healthier eating habits, which consequently results in a reduction in UPF consumption [132,133]. Sedentary individuals had a higher prevalence of consumption of all the types of food analyzed, with this effect being more evident for fast food. These findings corroborate previous studies that suggest an association between a sedentary lifestyle and less healthy eating patterns [134,135].

Individuals who were dissatisfied with their own bodies consumed more sweets, soft drinks and industrialized/ultra-processed salty foods. These findings reinforce the hypothesis that body dissatisfaction may be related to less healthy eating habits [136]. As mentioned, this may be due to emotional and psychological mechanisms, where individuals who are more dissatisfied with their body perception seek comfort in UPF [95,96]. Another strong association is the greater use of maladaptive eating styles, which include emotional eating, restrictive eating, food addiction, food neophobia and unhealthy weight control behaviors [137]. Other associations have also shown that body dissatisfaction is related to low self-esteem, anxiety and depression [138,139] which can lead to episodes of food compensation and the higher consumption of UPF [140,141].

Sugar-sweetened beverages are associated with the concomitant consumption of other UPF (such as fast food), as well as being risk factors for the development of chronic non-communicable diseases (CNCDs) [142,143,144]. On the other hand, in this study, television viewing time of three hours or more per day was associated with the lower consumption of soft drinks. This contradicts much of the literature, which generally points to a positive association between sedentary behavior and the intake of sugary drinks [142,145,146,147]. However, a similar result was observed by Jezewska-Zychowicz et al. (2018) [134] in Poland, where they found no association between watching TV and eating patterns such as fast food and sweets. The authors suggest that the consumption of certain foods can occur habitually and unrelated to specific behaviors, depending on the sociocultural context [134]. Thus, it is possible that among education workers, soft drink consumption is influenced by factors such as work environment, availability or already established habits and not necessarily by screen time. These findings reinforce the importance of considering cultural and environmental variables when interpreting behavioral and dietary associations.

### 4.3. Mental Health

The findings suggest a significant relationship between symptoms of stress, anxiety and depression and the consumption of UPF, especially sweets, soft drinks and industrialized/ultra-processed salty foods. Employees with higher levels of stress and anxiety had a higher prevalence of consumption of soft drinks and industrialized/ultra-processed salty foods. This reinforces the hypothesis that mental disorders may be associated with a greater search for hypercaloric and ultra-processed foods as a form of emotional regulation [6,70,148]. In addition, studies indicate a strong association between unhealthy eating and mental health [5,6,149], where high UPF intake can contribute to worsening mental symptoms [15,150] due to its impact on metabolism and brain neurochemistry [151].

Individuals with high levels of depression were up to 2.43 times more likely to consume salty ultra-processed foods on a regular basis, and the prevalence of their consumption of sweets and soft drinks was also high. This high consumption of UPFs was also associated with increased psychological distress (PRadj = 1.23; 95% CI = 1.10–1.38) as an indicator of depression over a 15-year period in a cohort study carried out in Melbourne [152]. A similar study in Brazil also found that participants in the highest quartile of UPF consumption had a higher risk of developing depression (PRadj = 1.82; 95% CI = 1.15–2.88) than those in the lowest quartile [153].

### 4.4. Quality of Life

Likewise, an inverse relationship was observed between quality of life and UPF consumption. Individuals with a poorer perception of quality of life, especially in the physical and psychological domains, had a higher consumption of sweets, soft drinks and salty industrialized/ultra-processed foods. Other studies also confirm our findings, where quality of life scores were lower when associated with the high consumption of UPF in different contexts [154,155,156,157]. Since the well-being of educators is directly associated with their ability to influence the nutritional satisfaction, health and well-being of students [158,159], they, in turn, are affected by harmful factors such as workload, the school environment and high responsibilities [9].

The social and environmental domains, as well as the total score, also had a significant relationship, especially with the consumption of industrialized/ultra-processed salty foods. The consumption of this food corresponded to a quality of life up to 1.99 times greater in individuals who were in the worst tertile evaluated. These findings reinforce the need for strategies that promote not only healthy eating but also interventions that improve the quality of life and mental well-being of education workers.

## 5. Limitations and Implications

This study has some limitations. The first is the cross-sectional design, which does not allow causality to be established between the regular consumption of UPF and associated factors. Secondly, it concerns the period during which the questionnaires were administered, which ranged from June to November 2022, going beyond the school term and school vacations, which may reflect, in some cases, different levels of stress, eating habits and work routines. In addition, the use of self-reported questionnaires for the assessments that were used may have been subject to response bias, as they depended on the participants’ memory. Finally, although the tool used is valid, the data may have been influenced by social desirability bias, in which participants reported healthier eating patterns than the real ones. This bias, coupled with limited recall and the timing of filling in the questionnaire, could affect the accuracy of the data and make comparisons between servers difficult.

However, the robust sample size and adjusted analysis make the findings more reliable. Furthermore, this is the first study to analyze the food consumption of civil servants from the federal education network. Given these results, it is essential that public policies and institutional actions are developed to promote healthy eating habits among education workers, taking into account individual, regional and occupational factors. Interventions aimed at improving quality of life and psychological support may be fundamental to reducing UPF consumption and its long-term impact on health.

## 6. Conclusions

In this study, we analyzed the factors associated with the consumption of UPF and their relationship with the quality of life and mental health of education workers. The data indicate that the regular consumption of soft drinks, sweets and industrialized salty snacks was associated with various factors, mainly sociodemographic, lifestyle habits, quality of life and mental health. Adopting healthier eating habits can improve general well-being, contributing to higher quality of life and greater productivity.

Given the cross-sectional design of the study, future research is recommended to adopt a longitudinal approach to exploring possible causal pathways between UPF consumption and mental health-related outcomes among education professionals. Additionally, future research should evaluate intersectoral interventions combining dietary guidance with stress management, sleep quality improvement and psychological support strategies. Implementing institutional health promotion programs that consider the specifics of the educational environment can significantly contribute to the overall well-being of these workers, positively impacting the quality of education provided.

## Figures and Tables

**Table 1:** Sociodemographic characteristics of the Brazilian federal education network employees surveyed (*n* = 1563).

	2022	
	*n*	%
Sex		
Male	668	42.7
Famale	895	57.3
Age group		
≤35	389	24.9
36–41	472	30.2
42–49	372	23.8
≥50	330	21.1
Marital status		
Married	1019	65
Single	377	24
Divorced/widowed	167	11
Level of education		
HS/HE/PTE	332	21.2
Specialist/MBA	484	31
Master’s	111	7.1
Doctorate/PhD	636	40.7
Region		
Center–West	504	32.2
North and Northeast	411	26.3
Southwest	406	26
South	242	15.5
Position		
ATE	878	56.2
Teacher	685	43.8
Length of service		
1–5 years	313	20
6–10 years	674	43.1
≥11 years	576	36.9
Region of residence		
Urban	1486	95.1
Rural	77	4.9

HS/HE/PTE: High School/Higher Education/Professional and Technological Education.

**Table 2:** Weekly consumption of ultra-processed foods according to gender and type of food, by the Brazilian federal education network employees surveyed (*n* = 1563).

Food/Weekly Frequency	Total		Male		Famale	
	%	*n*	%	*n*	%	*n*
Sweets						
Did not eat	13.2	206	15.7	105	11.3	101
1 day	16.4	257	17.5	117	15.6	140
2 days	19.7	308	20.5	137	19.1	171
3 days	16.1	252	16.9	113	15.5	139
4 days	9.3	145	8.7	58	9.7	87
5 days	7.5	117	6.4	43	8.3	74
6 days	4.9	76	4.2	28	5.4	48
7 days	12.9	202	10	67	15.1	135
Soft Drinks						
Did not eat	40.8	637	35.8	239	44.5	398
1 day	19.6	307	19.8	132	19.6	175
2 days	16.3	254	19.5	130	13.9	124
3 days	10.6	165	12	80	9.5	85
4 days	4.4	69	4.2	28	4.6	41
5 days	3.4	53	2.7	18	3.9	35
6 days	1.2	18	1.6	11	0.8	7
7 days	3.8	60	4.5	30	3.4	30
Industrialized/Ultra-Processed Salty Foods						
Did not eat	19.6	307	16.2	108	22.2	199
1 day	22.4	350	21	140	23.5	210
2 days	19.4	304	20.1	134	19	170
3 days	15.7	246	18.6	124	13.6	122
4 days	8.3	129	8.8	59	7.8	70
5 days	5.6	87	6.1	41	5.1	46
6 days	2.8	44	3.1	21	2.6	23
7 days	6.1	96	6.1	41	6.1	55
Fast Food						
Did not eat	40.6	635	37.9	253	42.7	382
1 day	30.4	475	30.4	203	30.4	272
2 days	16.8	262	18	120	15.9	142
3 days	7.4	115	8.4	56	6.6	59
4 days	2.3	36	3.3	22	1.6	14
5 days	1.3	21	1.2	8	1.5	13
6 days	0.4	7	0.3	2	0.6	5
7 days	0.8	12	0.6	4	0.9	8

**Table 3:** Adjusted analysis of the prevalence ratio of the regular consumption of ultra-processed foods by civil servants in the Brazilian federal education network (*n* = 1563).

	RC Sweets			RC Soft Drinks			RC Industrialized/Ultra-Processed Salty Foods			RC Fast Food		
	*n* (%)	PRadj (95% CI)	*p*	*n* (%)	PRadj (95% CI)	*p*	*n* (%)	PRadj (95% CI)	*p*	*n* (%)	PRadj (95% CI)	*p*
Sociodemographic												
Sex												
Male	138 (20.7)	1		59 (8.8)	1		103 (15.4)	1		14 (2.1)	1	
Female	257 (28.7)	1.40 (1.17–1.67)	<0.001	72 (8)	0.96 (0.69–1.33)	0.790	124 (13.9)	0.92 (0.72–1.18)	0.524	26 (2.9)	1.50 (0.78–2.88)	0.223
Age group												
22–34	105 (32.9)	1		27 (8.5)	1		56 (17.6)	1		10 (3.1)	1	
35–47	236 (28)	0.86 (0.70–1.06)	0.159	83 (9.9)	1.13 (0.73–1.74)	0.586	130 (15.4)	0.90 (0.66–1.22)	0.504	18 (2.1)	0.62 (0.26–1.46)	0.272
48–60	47 (13.5)	0.42 (0.30–0.58)	<0.001	15 (4.3)	0.48 (0.25–0.89)	0.020	34 (9.7)	0.56 (0.37–0.86)	0.008	12 (3.4)	0.86 (0.35–2.09)	0.739
61–72	7 (13.2)	0.37 (0.18–0.77)	0.008	6 (11.3)	0.14 (0.46–2.85)	0.779	7 (13.2)	0.69 (0.32–1.51)	0.352	-	-	-
Region												
North–Northeast	71 (17.3)	1		19 (4.6)	1		47 (11.4)	1		10 (2.4)	1	
South	73 (30.2)	1.89 (1.43–2.50)	<0.001	14 (5.8)	1.29 (0.66–2.55)	0.457	42 (17.4)	1.59 (1.08–2.36)	0.019	5 (2.1)	0.99 (0.34–2.88)	0.985
Midwest	123 (24.4)	1.47 (1.14–1.91)	0.003	56 (11.1)	2.46 (1.46–4.15)	0.001	72 (14.3)	1.28 (0.90–1.81)	0.167	17 (3.4)	1.47 (0.66–3.28)	0.351
Southeast	128 (31.5)	2.01 (1.57–2.59)	<0.001	42 (10.3)	2.37 (1.38–4.08)	0.002	66 (16.3)	1.50 (1.05–2.14)	0.025	8 (2)	0.89 (0.34–2.31)	0.811
Marital status												
Married	242 (23.7)	1		90 (8.8)	1		145 (14.2)	1		21 (2.1)	1	
Single	114 (30.2)	1.19 (0.98–1.43)	0.074	28 (7.4)	0.87 (0.58–1.31)	0.511	57 (15.1)	1.02 (0.77–1.38)	0.866	15 (4)	2.02 (1.05–3.88)	0.034
Divorced/widowed	39 (23.4)	1.05 (0.80–1.39)	0.705	13 (7.8)	1.01 (0.58–1.77)	0.966	25 (15)	1.12 (0.75–1.65)	0.582	4 (2.4)	1.01 (0.35–2.95)	0.979
Work and Training												
Level of education												
Doctorate/PHD	168 (26.4)	1		56 (8.8)	1		91 (14.3)	1		15 (2.4)	1	
Master’s degree	28 (25.2)	1.08 (0.75–1.56)	0.674	9 (8.1)	1.18 (0.58–2.41)	0.647	23 (20.7)	1.66 (1.07–2.59)	0.024	4 (3.6)	1.83 (0.55–6.09)	0.324
Specialization/MBA	122 (25.2)	1.03 (0.84–1.28)	0.757	42 (8.7)	1.15 (0.77–1.73)	0.498	71 (14.7)	1.14 (0.84–1.55)	0.387	11 (2.3)	1.07 (0.48–2.37)	0.867
HS/HE/PTE	77 (23.2)	0.80 (0.63–1.02)	0.073	24 (7.2)	0.73 (0.44–1.21)	0.224	42 (12.7)	0.83 (0.58–1.17)	0.286	10 (3)	1.01 (0.43–2.34)	0.983
Position												
ATE	215 (24.5)	1		71 (8.1)	1		125 (14.2)	1		20 (2.3)	1	
Teacher	180 (26.3)	1.45 (1.17–1.78)	0.001	60 (8.8)	1.43 (0.93–2.19)	0.108	102 (14.9)	1.39 (1.03–1.88)	0.029	20 (2.9)	1.48 (0.74–2.96)	0.265
Length of service												
≥11 years	119 (20.7)	1		44 (7.6)	1		71 (12.3)	1		18 (3.1)	1	
6–10 years	177 (26.3)	1.00 (0.81–1.24)	0.968	66 (9.8)	1.06 (0.73–1.53)	0.764	108 (16)	1.11 (0.83–1.49)	0.481	13 (1.9)	0.57 (0.28–1.19)	0.134
1–5 years	99 (31.6)	1.10 (0.85–1.42)	0.459	21 (6.7)	0.73 (0.43–1.25)	0.250	48 (15.3)	0.98 (0.67–1.41)	0.896	9 (2.9)	0.71 (0.32–1.59)	0.406
Body Perception and Lifestyle Habits												
Body satisfaction												
1° tercil—satisfied	181 (22.0)	1		56 (6.8)	1		92 (11.2)	1		26 (3.2)	1	
2° tercil—neutral	122 (25.8)	1.13 (0.93–1.37)	0.230	39 (8.3)	1.24 (0.83–1.84)	0.285	71 (15)	1.36 (1.02–1.82)	0.035	6 (1.3)	0.38 (0.16–0.93)	0.034
3° tercil—dissatisfied	92 (34.3)	1.39 (1.13–1.71)	0.002	36 (13.4)	1.90 (1.28–2.81)	0.001	64 (32.9)	2.07 (1.55–2.76)	<0.001	8 (3.0)	0.88 (0.41–1.91)	0.756
Quality of sleep												
1° tercil—satisfied	172 (23.3)	1		52 (7)	1		90 (12.2)	1		16 (2.2)	1	
2° tercil—neutral	92 (22.8)	0.99 (0.80–1.23)	0.951	33 (8.2)	1.18 (0.78–1.78)	0.427	50 (12.4)	1.02 (0.74–1.41)	0.891	8 (2)	0.89 (0.39–2.05)	0.793
3° tercil—dissatisfied	131 (31)	1.26 (1.04–1.52)	0.017	46 (10.9)	1.48 (1.01–2.15)	0.042	87 (20.6)	1.64 (1.26–2.15)	<0.001	16 (3.8)	1.73 (0.88–3.37)	0.109
Hours of sleep												
7–8 h	204 (25)	1		64 (7.9)	1		103 (12.6)	1		17 (2.1)	1	
≥9 h	19 (31.1)	1.07 (0.73–1.58)	0.726	8 (13.1)	1.57 (0.79–3.13)	0.201	8 (13.1)	0.96 (0.49–1.87)	0.912	-	-	-
≤6 h	172 (25)	1.07 (0.90–1.27)	0.442	59 (8.6)	1.07 (0.77–1.50)	0.683	116 (16.9)	1.38 (1.08–1.77)	0.011	23 (3.3)	1.58 (0.83–3.00)	0.161
TV time (h/d)												
<1 h	144 (26.6)	1		55 (10.2)	1		72 (13.3)	1		14 (2.6)	1	
1–2 h	148 (24.7)	0.95 (0.79–1.16)	0.634	56 (9.4)	0.92 (0.65–1.30)	0.625	85 (14.2)	1.07 (0.80–1.44)	0.645	15 (2.5)	0.96 (0.47–1.95)	0.914
≥3 h	85 (24.9)	0.97 (0.78–1.21)	0.797	14 (4.1)	0.41 (0.23–0.73)	0.002	57 (16.7)	1.24 (0.90–1.71)	0.184	8 (2.3)	0.92 (0.39–2.19)	0.857
Regular physical activity												
Yes	187 (21.5)	1		43 (4.9)	1		96 (11)	1		13 (1.5)	1	
No	208 (30)	1.37 (1.16–1.62)	<0.001	88 (12.7)	2.42 (1.71–3.42)	<0.001	131 (18.9)	1.67 (1.31–2.14)	<0.001	27 (3.9)	2.67 (1.36–5.25)	0.004
Weekly frequency of physical activity												
≥4 days	63 (17.3)	1		14 (3.8)	1		34 (9.3)	1		6 (1.6)	1	
1–3 days	120 (24.7)	1.42 (1.09–1.85)	0.010	28 (5.8)	1.51 (0.81–2.81)	0.195	60 (12.4)	1.33 (0.90–1.98)	0.155	7 (1.4)	0.88 (0.30–2.63)	0.823
0 day	208 (30)	1.70 (1.33–2.18)	<0.001	88 (12.7)	3.14 (1.81–5.43)	<0.001	131 (18.9)	1.99 (1.39–2.84)	<0.001	27 (3.9)	2.43 (0.99–5.92)	0.051
Mental Health—DASS-21												
Stress												
Normal	187 (21.8)	1		51 (6)	1		94 (11)	1		17 (2)	1	
Moderate	116 (28.6)	1.14 (0.94–1.49)	0.184	40 (9.9)	1.57 (1.05–2.35)	0.028	62 (15.3)	1.36 (1.01–1.83)	0.045	13 (3.2)	1.55 (0.75–3.19)	0.239
High	92 (30.6)	1.21 (0.98–1.49)	0.078	40 (13.3)	2.11 (1.43–3.13)	<0.001	71 (14.5)	2.11 (1.58–2.80)	<0.001	10 (3.3)	1.58 (0.74–3.37)	0.232
Anxiety												
Normal	194 (22.4)	1		55 (6.4)	1		100 (11.5)	1		17 (2)	1	
Moderate	100 (25.3)	1.11 (0.91–1.36)	0.295	37 (10.2)	1.60 (1.08–2.36)	0.018	56 (15.5)	1.32 (0.97–1.78)	0.073	11 (27.5)	1.49 (0.71–3.13)	0.289
High	101 (30.1)	1.18 (0.96–1.45)	0.106	39 (11.6)	1.83 (1.24–2.70)	0.002	71 (21.1)	1.81 (1.36–2.41)	<0.001	12 (3.6)	1.65 (0.79–3.44)	0.181
Depression												
Normal	175 (21.1)	1		53 (6.4)	1		83 (10)	1		19 (2.3)	1	
Moderate	139 (30)	1.22 (1.04–1.53)	0.016	40 (8.6)	1.25 (0.85–1.86)	0.260	75 (16.2)	1.55 (1.16–2.08)	0.003	11 (2.4)	1.01 (0.48–2.12)	0.980
High	81 (30.2)	1.29 (1.04–1.60)	0.020	38 (14.2)	2.05 (1.39–3.03)	<0.001	69 (25.7)	2.43 (1.82–3.26)	<0.001	10 (3.7)	1.52 (0.72–3.18)	0.268
Quality of Life—WHOQOL-bref												
Physics												
3rd tertile—better	101 (22)	1		33 (7.2)	1		54 (11.7)	1		9 (2)	1	
2nd tertile	132 (24.5)	1.09 (0.88–1.37)	0.423	33 (6.1)	0.85 (0.54–1.36)	0.503	70 (13)	1.14 (0.82–1.60)	0.436	14 (2.6)	1.36 (0.59–3.14)	0.474
1st tertile—worse	162 (28.7)	1.27 (1.02–1.57)	0.030	65 (11.5)	1.62 (1.07–2.45)	0.021	103 (18.3)	1.62 (1.19–2.20)	0.002	17 (3)	1.46 (0.67–3.18)	0.335
Psychological												
3rd tertile—better	126 (19.5)	1		40 (6.2)	1		61 (9.4)	1		16 (2.5)	1	
2nd tertile	119 (26.7)	1.29 (1.04–1.60)	0.020	33 (7.4)	1.23 (0.79–1.93)	0.354	58 (13)	1.37 (0.98–1.93)	0.065	13 (2.9)	1.20 (0.57–2.52)	0.625
1st tertile—worse	150 (31.8)	1.44 (1.18–1.77)	<0.001	58 (12.3)	1.92 (1.31–2.82)	0.001	108 (22.9)	2.32 (1.73–3.11)	<0.001	11 (2.3)	0.93 (0.43–1.99)	0.852
Social												
3rd tertile—better	69 (24.4)	1		24 (8.5)	1		34 (12)	1		7 (2.5)	1	
2nd tertile	196 (26.8)	1.11 (0.88–1.40)	0.373	46 (6.3)	0.71 (0.45–1.14)	0.161	91 (12.4)	1.04 (0.72–1.51)	0.823	19 (2.6)	1.10 (0.46–2.63)	0.822
1st tertile—worse	130 (23.7)	0.99 (0.77–1.27)	0.934	61 (11.1)	1.27 (0.81–1.99)	0.301	102 (18.6)	1.55 (1.07–2.23)	0.019	14 (2.6)	1.09 (0.43–2.76)	0.862
Environmental												
3rd tertile—better	112 (25.6)	1		38 (8.7)	1		53 (12.1)	1		11 (2.5)	1	
2nd tertile	157 (26.2)	1.02 (0.83–1.25)	0.846	48 (8)	0.97 (0.64–1.46)	0.885	76 (12.7)	1.06 (0.76–1.47)	0.730	13 (2.2)	0.91 (0.40–2.07)	0.827
1st tertile—worse	126 (23.9)	0.95 (0.77–1.18)	0.657	45 (8.5)	1.05 (0.69–1.59)	0.818	98 (18.6)	1.56 (1.15–2.13)	0.005	16 (3)	1.35 (0.60–3.04)	0.473
Total score												
3rd tertile—better	113 (21.6)	1		32 (6.1)	1		55 (10.5)	1		13 (2.5)	1	
2nd tertile	139 (26.8)	1.22 (0.99–1.51)	0.059	41 (7.9)	1.34 (0.85–2.09)	0.202	62 (11.9)	1.14 (0.81–1.60)	0.448	11 (2.1)	0.88 (0.39–1.97)	0.753
1st tertile—worse	143 (27.4)	1.22 (0.99–1.51)	0.058	58 (11.1)	1.83 (1.21–2.77)	0.004	110 (21.1)	1.99 (1.48–2.69)	<0.001	16 (3.1)	1.24 (0.59–2.59)	0.568

Note: The “*n*” column represents absolute frequencies, while the “%” column represents relative frequencies. The analysis was conducted using the Poisson regression model with robust variance. The measure of effect was the PR with its respective 95% CI. The model was adjusted for confounding variables: gender, age group, level of education, region, job title and length of service. Higher scores on WHOQOL-bref indicated better quality of life. “HS/HE/PTE”: High School/Higher Education/Professional and Technological Education; ‘MBA’: Master’s in Business Administration.

## Data Availability

The data presented in this study are available on request from the corresponding author due to privacy.

## Supplementary Materials

The following supporting information can be downloaded at https://www.mdpi.com/article/10.3390/nu17152519/s1. Table S1: Results of the bivariate analysis of the prevalence ratio of regular consumption of ultra-processed foods by civil servants in the Brazilian federal education network (*n* = 1563).

## Abbreviations

The following abbreviations are used in this manuscript:

UPF	Ultra-Processed Food
RFEPCT	Federal Network of Professional, Scientific and Technological Education
PeNSE	National School Health Survey
DASS-21	Depression, Anxiety and Stress Scale
WHOQOL-bref	World Health Organization Quality of Life Brief Version
PRadj	Adjusted Prevalence Ratio
QoLE-BRA	Quality of Life in Brazilian Education
ICF	Informed Consent Form
MEC	Ministry of Education
CEFETs	Federal Technological Education Centers
PNP	Nilo Peçanha Platform
ATEs	Administrative Technicians in Education
IBGE	Instituto Brasileiro de Geografia e Estatística
CNCD	Chronic Non-Communicable Disease

## References

[1] Dai S., Wellens J., Yang N., Li D., Wang J., Wang L., Yuan S., He Y., Song P., Munger R. (2024). Ultra-Processed Foods and Human Health: An Umbrella Review and Updated Meta-Analyses of Observational Evidence. Clin. Nutr..

[2] Monteiro C.A., Cannon G., Moubarac J.-C., Levy R.B., Louzada M.L.C., Jaime P.C. (2018). The UN Decade of Nutrition, the NOVA Food Classification and the Trouble with Ultra-Processing. Public Health Nutr..

[3] Dicken S.J., Batterham R.L. (2024). Ultra-Processed Food and Obesity: What Is the Evidence?. Curr. Nutr. Rep..

[4] Juul F., Vaidean G., Parekh N. (2021). Ultra-Processed Foods and Cardiovascular Diseases: Potential Mechanisms of Action. Adv. Nutr..

[5] Mazloomi S.N., Talebi S., Mehrabani S., Bagheri R., Ghavami A., Zarpoosh M., Mohammadi H., Wong A., Nordvall M., Kermani M.A.H. (2023). The Association of Ultra-Processed Food Consumption with Adult Mental Health Disorders: A Systematic Review and Dose-Response Meta-Analysis of 260,385 Participants. Nutr. Neurosci..

[6] Ejtahed H.-S., Mardi P., Hejrani B., Mahdavi F.S., Ghoreshi B., Gohari K., Heidari-Beni M., Qorbani M. (2024). Association between Junk Food Consumption and Mental Health Problems in Adults: A Systematic Review and Meta-Analysis. BMC Psychiatry.

[7] Dicken S.J., Batterham R.L. (2021). The Role of Diet Quality in Mediating the Association between Ultra-Processed Food Intake, Obesity and Health-Related Outcomes: A Review of Prospective Cohort Studies. Nutrients.

[8] Martini D., Godos J., Bonaccio M., Vitaglione P., Grosso G. (2021). Ultra-Processed Foods and Nutritional Dietary Profile: A Meta-Analysis of Nationally Representative Samples. Nutrients.

[9] Jakstas T., Follong B., Bucher T., Miller A., Shrewsbury V.A., Collins C.E. (2023). Addressing Schoolteacher Food and Nutrition-Related Health and Wellbeing: A Scoping Review of the Food and Nutrition Constructs Used across Current Research. Int. J. Behav. Nutr. Phys. Act..

[10] Tsubono K., Ogawa M. (2022). The Analysis of Main Stressors among High-Stress Primary School Teachers by Job Positions: A Nationwide Survey in Japan. Front. Public Health.

[11] Hecht E.M., Rabil A., Martinez Steele E., Abrams G.A., Ware D., Landy D.C., Hennekens C.H. (2022). Cross-Sectional Examination of Ultra-Processed Food Consumption and Adverse Mental Health Symptoms. Public Health Nutr..

[12] Grajek M., Krupa-Kotara K., Białek-Dratwa A., Sobczyk K., Grot M., Kowalski O., Staśkiewicz W. (2022). Nutrition and Mental Health: A Review of Current Knowledge about the Impact of Diet on Mental Health. Front. Nutr..

[13] Lane M.M., Gamage E., Du S., Ashtree D.N., McGuinness A.J., Gauci S., Baker P., Lawrence M., Rebholz C.M., Srour B. (2024). Ultra-Processed Food Exposure and Adverse Health Outcomes: Umbrella Review of Epidemiological Meta-Analyses. BMJ.

[14] Monteiro C.A., Moubarac J.-C., Levy R.B., Canella D.S., Louzada M.L.d.C., Cannon G. (2018). Household Availability of Ultra-Processed Foods and Obesity in Nineteen European Countries. Public Health Nutr..

[15] Lopes Cortes M., Andrade Louzado J., Galvão Oliveira M., Moraes Bezerra V., Mistro S., Souto Medeiros D., Arruda Soares D., Oliveira Silva K., Nicolaevna Kochergin C., Honorato dos Santos de Carvalho V.C. (2021). Unhealthy Food and Psychological Stress: The Association between Ultra-Processed Food Consumption and Perceived Stress in Working-Class Young Adults. Int. J. Environ. Res. Public Health.

[16] Mattar J.B., Domingos A.L.G., Hermsdorff H.H.M., Juvanhol L.L., de Oliveira F.L.P., Pimenta A.M., Bressan J. (2022). Ultra-Processed Food Consumption and Dietary, Lifestyle and Social Determinants: A Path Analysis in Brazilian Graduates (CUME Project). Public Health Nutr..

[17] Bremner J., Moazzami K., Wittbrodt M., Nye J., Lima B., Gillespie C., Rapaport M., Pearce B., Shah A., Vaccarino V. (2020). Diet, Stress and Mental Health. Nutrients.

[18] Firth J., Gangwisch J.E., Borsini A., Wootton R.E., Mayer E.A. (2020). Food and Mood: How Do Diet and Nutrition Affect Mental Wellbeing?. BMJ.

[19] Monteiro C.A., Cannon G., Levy R.B., Moubarac J.-C., Louzada M.L., Rauber F., Khandpur N., Cediel G., Neri D., Martinez-Steele E. (2019). Ultra-Processed Foods: What They Are and How to Identify Them. Public Health Nutr..

[20] Tristan Asensi M., Napoletano A., Sofi F., Dinu M. (2023). Low-Grade Inflammation and Ultra-Processed Foods Consumption: A Review. Nutrients.

[21] Firth J., Veronese N., Cotter J., Shivappa N., Hebert J.R., Ee C., Smith L., Stubbs B., Jackson S.E., Sarris J. (2019). What Is the Role of Dietary Inflammation in Severe Mental Illness? A Review of Observational and Experimental Findings. Front. Psychiatry.

[22] Bekdash R.A. (2024). Epigenetics, Nutrition, and the Brain: Improving Mental Health through Diet. Int. J. Mol. Sci..

[23] Adan R.A.H., van der Beek E.M., Buitelaar J.K., Cryan J.F., Hebebrand J., Higgs S., Schellekens H., Dickson S.L. (2019). Nutritional Psychiatry: Towards Improving Mental Health by What You Eat. Eur. Neuropsychopharmacol..

[24] Głąbska D., Guzek D., Groele B., Gutkowska K. (2020). Fruit and Vegetable Intake and Mental Health in Adults: A Systematic Review. Nutrients.

[25] Hepsomali P., Groeger J.A. (2021). Diet, Sleep, and Mental Health: Insights from the UK Biobank Study. Nutrients.

[26] Houminer-Klepar N., Dopelt K. (2025). Associations Between Mediterranean Diet, Processed Food Consumption, and Symptoms of Anxiety and Depression: Cross-Sectional Study Among Israeli Adults. Foods.

[27] Firth J., Marx W., Dash S., Carney R., Teasdale S.B., Solmi M., Stubbs B., Schuch F.B., Carvalho A.F., Jacka F. (2019). The Effects of Dietary Improvement on Symptoms of Depression and Anxiety: A Meta-Analysis of Randomized Controlled Trials. Psychosom. Med..

[28] Gómez-Donoso C., Sánchez-Villegas A., Martínez-González M.A., Gea A., Mendonça R.d.D., Lahortiga-Ramos F., Bes-Rastrollo M. (2020). Ultra-Processed Food Consumption and the Incidence of Depression in a Mediterranean Cohort: The SUN Project. Eur. J. Nutr..

[29] Bizzozero-Peroni B., Martínez-Vizcaíno V., Fernández-Rodríguez R., Jiménez-López E., Núñez de Arenas-Arroyo S., Saz-Lara A., Díaz-Goñi V., Mesas A.E. (2025). The Impact of the Mediterranean Diet on Alleviating Depressive Symptoms in Adults: A Systematic Review and Meta-Analysis of Randomized Controlled Trials. Nutr. Rev..

[30] Dopelt K., Houminer-Klepar N. (2025). The Impact of Social Media on Disordered Eating: Insights from Israel. Nutrients.

[31] Ministério da Saúde (2014). Guia Alimentar Para a População Brasileira.

[32] Zapata M.E., Cediel G., Arrieta E., Rovirosa A., Carmuega E., Monteiro C.A. (2023). Ultra-Processed Foods Consumption and Diet Quality among Preschool Children and Women of Reproductive Age from Argentina. Public Health Nutr..

[33] Amorim A., Barbosa A.d.H., Sobral P.J.d.A. (2022). Hunger, Obesity, Public Policies, and Food-Based Dietary Guidelines: A Reflection Considering the Socio-Environmental World Context. Front. Nutr..

[34] Pulker C.E., Scott J.A., Pollard C.M. (2018). Ultra-Processed Family Foods in Australia: Nutrition Claims, Health Claims and Marketing Techniques. Public Health Nutr..

[35] Fan W., Lam J., Moen P., Kelly E., King R., McHale S. (2015). Constrained Choices? Linking Employees’ and Spouses’ Work Time to Health Behaviors. Soc. Sci. Med..

[36] Parker E.A., Feinberg T.M., Lane H.G., Deitch R., Zemanick A., Saksvig B.I., Turner L., Hager E.R. (2020). Diet Quality of Elementary and Middle School Teachers Is Associated with Healthier Nutrition-Related Classroom Practices. Prev. Med. Rep..

[37] Aydin G., Margerison C., Worsley A., Booth A. (2021). Parents’ and Teachers’ Views of the Promotion of Healthy Eating in Australian Primary Schools. BMC Public Health.

[38] Isaksen I.M., Dankel S.N. (2023). Ultra-Processed Food Consumption and Cancer Risk: A Systematic Review and Meta-Analysis. Clin. Nutr..

[39] Wang Z., Lu C., Cui L., Fenfen E., Shang W., Wang Z., Song G., Yang K., Li X. (2024). Consumption of Ultra-Processed Foods and Multiple Health Outcomes: An Umbrella Study of Meta-Analyses. Food Chem..

[40] Mendoza K., Smith-Warner S.A., Rossato S.L., Khandpur N., Manson J.E., Qi L., Rimm E.B., Mukamal K.J., Willett W.C., Wang M. (2024). Ultra-Processed Foods and Cardiovascular Disease: Analysis of Three Large US Prospective Cohorts and a Systematic Review and Meta-Analysis of Prospective Cohort Studies. Lancet Reg. Health Am..

[41] López-Olivares M., De Teresa Galván C., Nestares T., Fernández-Gómez E., Enrique-Mirón C. (2021). Lifestyle Factors Influencing Dietary Patterns of University Professors. Int. J. Environ. Res. Public Health.

[42] Uvacsek M., Simkó G., Boda-Ujlaky J., Kneffel Z. (2022). Frequency of Breakfast Eating and Obesity Prevalence in Primary School Teachers. Int. J. Environ. Res. Public Health.

[43] Silva J.P.d., Fischer F.M. (2020). Understudied School Teachers’ Work/Life Balance and Everyday Life Typologies. Chronobiol. Int..

[44] Almhdawi K.A., Obeidat D., Kanaan S.F., Hajela N., Bsoul M., Arabiat A., Alazrai A., Jaber H., Alrabbaie H. (2021). University Professors’ Mental and Physical Well-Being during the COVID-19 Pandemic and Distance Teaching. Work.

[45] Hibbs-Shipp S.K., Milholland M., Bellows L. (2015). Barriers and Facilitators to Healthy Eating and Activity in Head Start Staff: An Opportunity for Worksite Wellness. Am. J. Health Educ..

[46] Freitas A.M.C., Araújo T.M.d., Fischer F.M. (2020). Psychosocial Aspects at Work and the Quality of Sleep of Professors in Higher Education. Arch. Environ. Occup. Health.

[47] Hamilton L., Goodman L., Roberts L., Dial L.A., Pratt M., Musher-Eizenman D. (2021). Teacher Experience, Personal Health, and Dieting Status Is Associated with Classroom Health-Related Practices and Modeling. J. Sch. Health.

[48] Marangoni F., Martini D., Scaglioni S., Sculati M., Donini L.M., Leonardi F., Agostoni C., Castelnuovo G., Ferrara N., Ghiselli A. (2019). Snacking in Nutrition and Health. Int. J. Food Sci. Nutr..

[49] Vajdi M., Farhangi M.A. (2020). A Systematic Review of the Association between Dietary Patterns and Health-Related Quality of Life. Health Qual. Life Outcomes.

[50] Zhu Z., Chang Y. (2025). The Influence of Work Stress on the Work Performance of University Teachers in Zhejiang Province, China: The Mediating Role of Work Happiness. J. Educ. Social. Res..

[51] Wartenberg G., Aldrup K., Grund S., Klusmann U. (2023). Satisfied and High Performing? A Meta-Analysis and Systematic Review of the Correlates of Teachers’ Job Satisfaction. Educ. Psychol. Rev..

[52] Trisal A., Singh I., Garg G., Jorwal K., Singh A.K. (2025). Gut–Brain Axis and Brain Health: Modulating Neuroinflammation, Cognitive Decline, and Neurodegeneration. 3 Biotech.

[53] Wang D., Stewart D., Yuan Y., Chang C. (2015). Do Health-Promoting Schools Improve Nutrition in China?. Health Promot. Int..

[54] Perikkou A., Gavrieli A., Kougioufa M.-M., Tzirkali M., Yannakoulia M. (2013). A Novel Approach for Increasing Fruit Consumption in Children. J. Acad. Nutr. Diet..

[55] Oliveira I.F.R.d., Pereira N.G., Monteiro L.F., Rezende L.M.T.d., Lira C.A.B.d., Monfort-Pañego M., Costa W.P.d., Noll P.R.E.S., Noll M. (2025). Factors Influencing the Quality of Life and Mental Health of Brazilian Federal Education Network Employees: An Epidemiological Cross-Sectional Study. Heliyon.

[56] Brasil Lei No 11.892, de 29 de Dezembro de 2008 (2008). Institui a Rede Federal de Educação Profissional, Científica e Tecnológica, Cria Os Institutos Federais de Educação, Ciência e Tecnologia, e Dá Outras Providências. https://www.planalto.gov.br/ccivil_03/_ato2007-2010/2008/lei/l11892.htm.

[57] Galvão T., Tavares K.A., Fernandes M., Souza J.C.M.d., Noll M. (2021). Case Report: Scientific Dissemination as a Mediator of the Research Consolidation Process: Proposals for Institutionalization. F1000Research.

[58] Galvão T., Rayanne E., Silva Noll P., Aparecida Silveira E., Noll M. (2023). Fake News and Misinformation in Brazil: Critical Analyses Regarding Scientific Information in Pandemic Times. J. Hum. Growth Dev..

[59] Melo A.F., da Costa W.P., Rodrigues R.R., Nunes L.d.A.C.B., Noll P.R.E.S., Noll M. (2023). Panorama of Undergraduate Research in Brazil: Profile, Scientific Production, and Perceptions. Publications.

[60] Plataforma Nilo Peçanha. https://www.gov.br/mec-divulga-mapa-das-mais-de-7-mil-formaturas-antecipadas-de-cursos-da-saude/pt-br/pnp.

[61] Costa W.P.d., Walker A.F.M., Miñana-Signes V., Monfort-Pañego M., Noll P.R.E.S., Noll M. (2025). Scientific Output of Undergraduate Research Supervisors: The Role of Sex-Related, Academic Career, and Subject Areas in a Brazilian Public Institution. Front. Educ..

[62] Santos de Holanda Vieira V., Maria de Lima A. (2024). Technical-Administrative Servers in Education at Federal Institutes: A Study of Scientific Production in Brazil in Recent Years (2017–2022). Rev. Bras. Educ. Prof. Tecnol..

[63] Fleck M.P.d.A. (2000). The World Health Organization Instrument to Evaluate Quality of Life (WHOQOL-100): Characteristics and Perspectives. Cien Saude Colet..

[64] Power M., Bullinger M., Harper A. (1999). The World Health Organization WHOQOL-100: Tests of the Universality of Quality of Life in 15 Different Cultural Groups Worldwide. Health Psychol..

[65] Skevington S.M., Lotfy M., O’Connell K.A. (2004). The World Health Organization’s WHOQOL-BREF Quality of Life Assessment: Psychometric Properties and Results of the International Field Trial. A Report from the WHOQOL Group. Qual. Life Res..

[66] Lovibond P.F., Lovibond S.H. (1995). The Structure of Negative Emotional States: Comparison of the Depression Anxiety Stress Scales (DASS) with the Beck Depression and Anxiety Inventories. Behav. Res. Ther..

[67] Lovibond S.H., Lovibond P.F. (2011). Depression Anxiety Stress Scales. PsycTESTS Dataset.

[68] Wittayapun Y., Summart U., Polpanadham P., Direksunthorn T., Paokanha R., Judabood N., A’la M.Z. (2023). Validation of Depression, Anxiety, and Stress Scales (DASS-21) among Thai Nursing Students in an Online Learning Environment during the COVID-19 Outbreak: A Multi-Center Study. PLoS ONE.

[69] Makara-Studzińska M., Tyburski E., Załuski M., Adamczyk K., Mesterhazy J., Mesterhazy A. (2022). Confirmatory Factor Analysis of Three Versions of the Depression Anxiety Stress Scale (DASS-42, DASS-21, and DASS-12) in Polish Adults. Front. Psychiatry.

[70] Hajmir M.M., Shiraseb F., Ebrahimi S., Noori S., Ghaffarian-Ensaf R., Mirzaei K. (2022). Interaction between Ultra-Processed Food Intake and Genetic Risk Score on Mental Health and Sleep Quality. Eat. Weight. Disord. Stud. Anorex. Bulim. Obes..

[71] Al Saadi T., Zaher Addeen S., Turk T., Abbas F., Alkhatib M. (2017). Psychological Distress among Medical Students in Conflicts: A Cross-Sectional Study from Syria. BMC Med. Educ..

[72] Abdel Wahed W.Y., Hassan S.K. (2017). Prevalence and Associated Factors of Stress, Anxiety and Depression among Medical Fayoum University Students. Alex. J. Med..

[73] Taheri M., Esmaeili A., Irandoust K., Mirmoezzi M., Souissi A., Laher I., Dergaa I., Zouhal H. (2023). Mental Health, Eating Habits and Physical Activity Levels of Elite Iranian Athletes during the COVID-19 Pandemic. Sci. Sports.

[74] Ministério da Economia—Instituto Brasileiro de Geografia e Estatística—IBGE (2022). Pesquisa Nacional de Saúde do Escolar.

[75] Instituto Brasileiro de Geografia e Estatística—IBGE (2021). Pesquisa Nacional de Saúde do Escolar: 2019.

[76] Boklis-Berer M., Rauber F., Azeredo C.M., Levy R.B., Louzada M.L.d.C. (2021). School Meals Consumption Is Associated with a Better Diet Quality of Brazilian Adolescents: Results from the PeNSE 2015 Survey. Public Health Nutr..

[77] Noll P.R.e.S., Noll M., de Abreu L.C., Baracat E.C., Silveira E.A., Sorpreso I.C.E. (2019). Ultra-Processed Food Consumption by Brazilian Adolescents in Cafeterias and School Meals. Sci. Rep..

[78] Domingues J.G., Santos F.S.d., Kaufmann C.C., Muniz L.C., Bielemann R.M., Mintem G.C. (2023). Healthy Eating Markers among Adolescents from the Municipal School System in Pelotas, Rio Grande Do Sul, Brazil, 2019: A Cross-Sectional Study. Epidemiol. Serviços Saúde.

[79] (2022). Ministério Da Saúde Vigitel Brazil 2006–2021: Surveillance of Risk and Protective Factors for Chronic Diseases by Telephone Survey: Estimates of Frequency and Sociodemographic Distribution of Nutritional Status and Food Consumption in the Capitals of the 26 Brazilian States and the Federal District between 2006 and 2021: Nutritional Status and Food Consumption; Brasília. http://bvsms.saude.gov.br/bvs/publicacoes/vigitel_brasil_2006-2021_estado_nutricional.pdf.

[80] de Souza A.L.G., de Almeida A.A., Noll P.R.E.S., Noll M. (2021). Unhealthy Life Habits Associated with Self-Induced Vomiting and Laxative Misuse in Brazilian Adolescents. Sci. Rep..

[81] Ventura Barbosa Gonçalves H., Canella D.S., Bandoni D.H. (2020). Temporal Variation in Food Consumption of Brazilian Adolescents (2009–2015). PLoS ONE.

[82] Locatelli N.T., Canella D.S., Bandoni D.H. (2018). Positive Influence of School Meals on Food Consumption in Brazil. Nutrition.

[83] Azeredo C.M., de Rezende L.F.M., Canella D.S., Moreira Claro R., de Castro I.R.R., Luiz O.d.C., Levy R.B. (2015). Dietary Intake of Brazilian Adolescents. Public Health Nutr..

[84] Machado C.T., Ferreira L.S., Cezar T.T. (2021). Challenges of Teachers of Basic Technical and Technological Education in Brazil. Educ. Policy Anal. Arch..

[85] Ramos L.d.F.d.C., Macêdo K.B. (2018). Reflections on the Occupational Illnesses of Technical-Administrativeemployees in Education. Argumentum.

[86] Sun G.-W., Shook T.L., Kay G.L. (1996). Inappropriate Use of Bivariable Analysis to Screen Risk Factors for Use in Multivariable Analysis. J. Clin. Epidemiol..

[87] Barros A.J., Hirakata V.N. (2003). Alternatives for Logistic Regression in Cross-Sectional Studies: An Empirical Comparison of Models That Directly Estimate the Prevalence Ratio. BMC Med. Res. Methodol..

[88] Park S., Xu F., Town M., Blanck H.M. (2016). Prevalence of Sugar-Sweetened Beverage Intake Among Adults—23 States and the District of Columbia, 2013. MMWR Morb. Mortal. Wkly. Rep..

[89] Vandevijvere S., De Ridder K., Fiolet T., Bel S., Tafforeau J. (2019). Consumption of Ultra-Processed Food Products and Diet Quality among Children, Adolescents and Adults in Belgium. Eur. J. Nutr..

[90] Park S., Lee S.H., Blanck H.M. (2023). Characteristics Associated with Being a High Consumer of Sweet Foods and Sugar-Sweetened Beverages among US Adults during the COVID-19 Pandemic, 2021. Nutrients.

[91] Bueno M.B., Marchioni D.M.L., César C.L.G., Fisberg R.M. (2012). Added Sugars: Consumption and Associated Factors among Adults and the Elderly. São Paulo, Brazil. Rev. Bras. Epidemiol..

[92] Benajiba N., Bernstein J., Aboul-Enein B.H. (2020). Attitudes toward Sweetened Soft Drinks and Consumption Patterns among Saudi Women: A Cross-Sectional Study. Eat. Behav..

[93] Rauber F., Steele E.M., Louzada M.L.d.C., Millett C., Monteiro C.A., Levy R.B. (2020). Ultra-Processed Food Consumption and Indicators of Obesity in the United Kingdom Population (2008–2016). PLoS ONE.

[94] Silva Júnior J.N.B.d., Freiria C.N., Silva G.M.d., Corona L.P. (2023). Factors Associated with Added Sugar Consumption of Older Adults from the Region of Campinas-SP, Brazil. Cien Saude Colet..

[95] Malo-Vintimilla L., Aguirre C., Vergara A., Fernández-Verdejo R., Galgani J.E. (2024). Resting Energy Metabolism and Sweet Taste Preference during the Menstrual Cycle in Healthy Women. Br. J. Nutr..

[96] Lampuré A., Schlich P., Deglaire A., Castetbon K., Péneau S., Hercberg S., Méjean C. (2015). Sociodemographic, Psychological, and Lifestyle Characteristics Are Associated with a Liking for Salty and Sweet Tastes in French Adults. J. Nutr..

[97] Tsuno K., Kawachi I., Inoue A., Nakai S., Tanigaki T., Nagatomi H., Kawakami N. (2019). Long Working Hours and Depressive Symptoms: Moderating Effects of Gender, Socioeconomic Status, and Job Resources. Int. Arch. Occup. Environ. Health.

[98] Dinh H., Strazdins L., Welsh J. (2017). Hour-Glass Ceilings: Work-Hour Thresholds, Gendered Health Inequities. Soc. Sci. Med..

[99] Monteiro C.A., Moubarac J.-C., Cannon G., Ng S.W., Popkin B. (2013). Ultra-processed Products Are Becoming Dominant in the Global Food System. Obes. Rev..

[100] Juul F., Martinez-Steele E., Parekh N., Monteiro C.A., Chang V.W. (2018). Ultra-Processed Food Consumption and Excess Weight among US Adults. Br. J. Nutr..

[101] Juul F., Parekh N., Martinez-Steele E., Monteiro C.A., Chang V.W. (2022). Ultra-Processed Food Consumption among US Adults from 2001 to 2018. Am. J. Clin. Nutr..

[102] Kliemann N., Al Nahas A., Vamos E.P., Touvier M., Kesse-Guyot E., Gunter M.J., Millett C., Huybrechts I. (2022). Ultra-Processed Foods and Cancer Risk: From Global Food Systems to Individual Exposures and Mechanisms. Br. J. Cancer.

[103] Andrade G.C., Gombi-Vaca M.F., Louzada M.L.d.C., Azeredo C.M., Levy R.B. (2020). The Consumption of Ultra-Processed Foods According to Eating out Occasions. Public Health Nutr..

[104] Bertoni Maluf V.A., Bucher Della Torre S., Jotterand Chaparro C., Belle F.N., Khalatbari-Soltani S., Kruseman M., Marques-Vidal P., Chatelan A. (2022). Description of Ultra-Processed Food Intake in a Swiss Population-Based Sample of Adults Aged 18 to 75 Years. Nutrients.

[105] Marchese L., Livingstone K.M., Woods J.L., Wingrove K., Machado P. (2022). Ultra-Processed Food Consumption, Socio-Demographics and Diet Quality in Australian Adults. Public Health Nutr..

[106] Levy R.B., Andrade G.C., Cruz G.L.d., Rauber F., Louzada M.L.d.C., Claro R.M., Monteiro C.A. (2022). Three Decades of Household Food Availability According to NOVA–Brazil, 1987–2018. Rev. Saude Publica.

[107] Louzada M.L.d.C., Cruz G.L.d., Silva K.A.A.N., Grassi A.G.F., Andrade G.C., Rauber F., Levy R.B., Monteiro C.A. (2023). Consumption of Ultra-Processed Foods in Brazil: Distribution and Temporal Evolution 2008–2018. Rev. Saude Publica.

[108] Sung H., Park J.M., Oh S.U., Ha K., Joung H. (2021). Consumption of Ultra-Processed Foods Increases the Likelihood of Having Obesity in Korean Women. Nutrients.

[109] Sandri E., Pérez-Bermejo M., Cabo A., Cerdá-Olmedo G. (2023). Living Alone: Associations with Diet and Health in the Spanish Young Adult Population. Nutrients.

[110] Almoraie N.M., Alothmani N.M., Alomari W.D., Al-amoudi A.H. (2024). Addressing Nutritional Issues and Eating Behaviours among University Students: A Narrative Review. Nutr. Res. Rev..

[111] Higgs S., Thomas J. (2016). Social Influences on Eating. Curr. Opin. Behav. Sci..

[112] Jönsson H., Michaud M., Neuman N. (2021). What Is Commensality? A Critical Discussion of an Expanding Research Field. Int. J. Environ. Res. Public Health.

[113] Rosmiati R., Reswari Haryana N., Firmansyah H., Purba R., Rahman Nurfazriah L., Edwin Fransiari M. (2022). The Relationship between Occupational Stress and Diet Quality with Productivity Loss in Islamic School Teachers in Medan: A Cross-Sectional Study. BIO Web Conf..

[114] Ramberg J., Brolin Låftman S., Åkerstedt T., Modin B. (2020). Teacher Stress and Students’ School Well-Being: The Case of Upper Secondary Schools in Stockholm. Scand. J. Educ. Res..

[115] Amaro J.M.R.S., Dumith S.C. (2018). Excessive Daytime Sleepiness and Quality of Life Related to the Health of University Professors. J. Bras. Psiquiatr..

[116] Grosso G., Godos J., Micek A. (2022). Ultra-Processed Food Intake Is Associated with Worse Mental Health in Southern Italian Individuals. Eur. J. Public Health.

[117] Duquenne P., Capperella J., Fezeu L.K., Srour B., Benasi G., Hercberg S., Touvier M., Andreeva V.A., St-Onge M.-P. (2024). The Association Between Ultra-Processed Food Consumption and Chronic Insomnia in the NutriNet-Santé Study. J. Acad. Nutr. Diet..

[118] Pourmotabbed A., Awlqadr F.H., Mehrabani S., Babaei A., Wong A., Ghoreishy S.M., Talebi S., Kermani M.A.H., Jalili F., Moradi S. (2024). Ultra-Processed Food Intake and Risk of Insomnia: A Systematic Review and Meta-Analysis. Nutrients.

[119] Klok M.D., Jakobsdottir S., Drent M.L. (2007). The Role of Leptin and Ghrelin in the Regulation of Food Intake and Body Weight in Humans: A Review. Obes. Rev..

[120] Zuraikat F.M., Makarem N., Liao M., St-Onge M., Aggarwal B. (2020). Measures of Poor Sleep Quality Are Associated With Higher Energy Intake and Poor Diet Quality in a Diverse Sample of Women From the Go Red for Women Strategically Focused Research Network. J. Am. Heart Assoc..

[121] Bombarda A., St-Onge M., Seixas A., Williams N., Jean-Louis G., Killgore W.D., Wills C.C., Grandner M.A. (2020). 0235 Sleep Duration and Timing Associated with Eating Behaviors: Data from NHANES 2015–2016. Sleep.

[122] Baglioni C., Battagliese G., Feige B., Spiegelhalder K., Nissen C., Voderholzer U., Lombardo C., Riemann D. (2011). Insomnia as a Predictor of Depression: A Meta-Analytic Evaluation of Longitudinal Epidemiological Studies. J. Affect. Disord..

[123] Riemann D. (2003). Primary Insomnia: A Risk Factor to Develop Depression?. J. Affect. Disord..

[124] Maisey G., Cattani M., Devine A., Lo J., Fu S.C., Dunican I.C. (2022). Digging for Data: How Sleep Is Losing out to Roster Design, Sleep Disorders, and Lifestyle Factors. Appl. Ergon..

[125] Huang T., Mariani S., Redline S. (2020). Sleep Irregularity and Risk of Cardiovascular Events. J. Am. Coll. Cardiol..

[126] Barbosa S.S., Sousa L.C.M., de Oliveira Silva D.F., Pimentel J.B., Evangelista K.C.M.d.S., Lyra C.d.O., Lopes M.M.G.D., Lima S.C.V.C. (2022). A Systematic Review on Processed/Ultra-Processed Foods and Arterial Hypertension in Adults and Older People. Nutrients.

[127] Liu Y., Wheaton A.G., Chapman D.P., Cunningham T.J., Lu H., Croft J.B. (2016). Prevalence of Healthy Sleep Duration among Adults—United States, 2014. MMWR Morb. Mortal. Wkly. Rep..

[128] Arocha Rodulfo J.I. (2019). Sedentary Lifestyle a Disease from Xxi Century. Clín. Investig. Arterioscler..

[129] Jeong S.-W., Kim S.-H., Kang S.-H., Kim H.-J., Yoon C.-H., Youn T.-J., Chae I.-H. (2019). Mortality Reduction with Physical Activity in Patients with and without Cardiovascular Disease. Eur. Heart J..

[130] Posadzki P., Pieper D., Bajpai R., Makaruk H., Könsgen N., Neuhaus A.L., Semwal M. (2020). Exercise/Physical Activity and Health Outcomes: An Overview of Cochrane Systematic Reviews. BMC Public Health.

[131] Chastin S.F.M., Abaraogu U., Bourgois J.G., Dall P.M., Darnborough J., Duncan E., Dumortier J., Pavón D.J., McParland J., Roberts N.J. (2021). Effects of Regular Physical Activity on the Immune System, Vaccination and Risk of Community-Acquired Infectious Disease in the General Population: Systematic Review and Meta-Analysis. Sports Med..

[132] Brace A.M., De Andrade F.C., Finkelstein B. (2018). Assessing the Effectiveness of Nutrition Interventions Implemented among US College Students to Promote Healthy Behaviors: A Systematic Review. Nutr. Health.

[133] Nardocci M., Leclerc B.-S., Louzada M.-L., Monteiro C.A., Batal M., Moubarac J.-C. (2019). Consumption of Ultra-Processed Foods and Obesity in Canada. Can. J. Public Health.

[134] Jezewska-Zychowicz M., Gębski J., Guzek D., Świątkowska M., Stangierska D., Plichta M., Wasilewska M. (2018). The Associations between Dietary Patterns and Sedentary Behaviors in Polish Adults (LifeStyle Study). Nutrients.

[135] Martínez Steele E., Juul F., Neri D., Rauber F., Monteiro C.A. (2019). Dietary Share of Ultra-Processed Foods and Metabolic Syndrome in the US Adult Population. Prev. Med..

[136] Rounsefell K., Gibson S., McLean S., Blair M., Molenaar A., Brennan L., Truby H., McCaffrey T.A. (2020). Social Media, Body Image and Food Choices in Healthy Young Adults: A Mixed Methods Systematic Review. Nutr. Diet..

[137] Eck K.M., Quick V., Byrd-Bredbenner C. (2022). Body Dissatisfaction, Eating Styles, Weight-Related Behaviors, and Health among Young Women in the United States. Nutrients.

[138] Shagar P.S., Harris N., Boddy J., Donovan C.L. (2017). The Relationship Between Body Image Concerns and Weight-Related Behaviours of Adolescents and Emerging Adults: A Systematic Review. Behav. Change.

[139] Bornioli A., Lewis-Smith H., Slater A., Bray I. (2021). Body Dissatisfaction Predicts the Onset of Depression among Adolescent Females and Males: A Prospective Study. J. Epidemiol. Community Health.

[140] Oliveira N., Coelho G.M.d.O., Cabral M.C., Bezerra F.F., Faerstein E., Canella D.S. (2020). Association of Body Image (Dis)Satisfaction and Perception with Food Consumption According to the NOVA Classification: Pró-Saúde Study. Appetite.

[141] Minari T.P., Araújo-Filho G.M.d., Tácito L.H.B., Yugar L.B.T., Rubio T.d.A., Pires A.C., Vilela-Martin J.F., Cosenso-Martin L.N., Fattori A., Yugar-Toledo J.C. (2024). Effects of Mindful Eating in Patients with Obesity and Binge Eating Disorder. Nutrients.

[142] Boulos R., Vikre E.K., Oppenheimer S., Chang H., Kanarek R.B. (2012). ObesiTV: How Television Is Influencing the Obesity Epidemic. Physiol. Behav..

[143] Mansouri M., Sharifi F., Yaghubi H., Varmaghani M., Tabrizi Y.M., Nasiri M., Sadeghi O. (2020). Sugar-Sweetened Beverages Consumption in Relation to Hypertension among Iranian University Students: The MEPHASOUS Study. Eat. Weight Disord. Stud. Anorex. Bulim. Obes..

[144] Tapanee P., Reeder N., Christensen R., Tolar-Peterson T. (2023). Sugar, Non-Nutritive Sweetener Intake and Obesity Risk in College Students. J. Am. Coll. Health.

[145] Liang T., Kuhle S., Veugelers P.J. (2009). Nutrition and Body Weights of Canadian Children Watching Television and Eating While Watching Television. Public Health Nutr..

[146] Silva D.C.G.d., Segheto W., Amaral F.C.d.S., Reis N.d.A., Veloso G.S.S., Pessoa M.C., Novaes J.F.d., Longo G.Z. (2019). Consumption of Sweetened Beverages and Associated Factors in Adults. Cien Saude Colet..

[147] Costa C.d.S., Wendt A., Machado A.K.F., Ricardo L.I.C., Werneck A.d.O., Louzada M.L.d.C. (2025). Ultra-Processed Food Consumption Is Related to Screen Time among Brazilian Adolescents, Adults and Older Adults. Br. J. Nutr..

[148] Ulrich-Lai Y.M., Fulton S., Wilson M., Petrovich G., Rinaman L. (2015). Stress Exposure, Food Intake and Emotional State. Stress.

[149] Ghernati L., Tamim H., Chokor F.A.Z., Taktouk M., Assi B., Nasreddine L., Elbejjani M. (2025). Processed and Ultra-Processed Foods Are Associated with Depression and Anxiety Symptoms in a Cross-Sectional Sample of Urban Lebanese Adults. Nutr. Res..

[150] Hill D., Conner M., Clancy F., Moss R., Wilding S., Bristow M., O’Connor D.B. (2022). Stress and Eating Behaviours in Healthy Adults: A Systematic Review and Meta-Analysis. Health Psychol. Rev..

[151] O’Connor D.B., Thayer J.F., Vedhara K. (2021). Stress and Health: A Review of Psychobiological Processes. Annu. Rev. Psychol..

[152] Lane M.M., Lotfaliany M., Hodge A.M., O’Neil A., Travica N., Jacka F.N., Rocks T., Machado P., Forbes M., Ashtree D.N. (2023). High Ultra-Processed Food Consumption Is Associated with Elevated Psychological Distress as an Indicator of Depression in Adults from the Melbourne Collaborative Cohort Study. J. Affect. Disord..

[153] Leal A.C.G., Lopes L.J., Rezende-Alves K., Bressan J., Pimenta A.M., Hermsdorff H.H.M. (2023). Ultra-Processed Food Consumption Is Positively Associated with the Incidence of Depression in Brazilian Adults (CUME Project). J. Affect. Disord..

[154] Noll P.R.e.S., Noll M., Zangirolami-Raimundo J., Baracat E.C., Louzada M.L.d.C., Soares Júnior J.M., Sorpreso I.C.E. (2022). Life Habits of Postmenopausal Women: Association of Menopause Symptom Intensity and Food Consumption by Degree of Food Processing. Maturitas.

[155] Cadenhead J.W., Martínez-Steele E., Contento I., Kushi L.H., Lee A.R., Nguyen T.T.T., Lebwohl B., Green P.H.R., Wolf R.L. (2023). Diet Quality, Ultra-processed Food Consumption, and Quality of Life in a Cross-sectional Cohort of Adults and Teens with Celiac Disease. J. Hum. Nutr. Diet..

[156] Hosseininasab D., Shiraseb F., Bahrampour N., da Silva A., Hajinasab M.M., Bressan J., Mirzaei K. (2024). Ultra-Processed Food Consumption and Quality of Life: A Cross-Sectional Study in Iranian Women. Front. Public Health.

[157] Hosseinpour-Niazi S., Niknam M., Amiri P., Mirmiran P., Ainy E., Izadi N., Gaeini Z., Azizi F. (2024). The Association between Ultra-Processed Food Consumption and Health-Related Quality of Life Differs across Lifestyle and Socioeconomic Strata. BMC Public Health.

[158] Carroll A., York A., Fynes-Clinton S., Sanders-O’Connor E., Flynn L., Bower J.M., Forrest K., Ziaei M. (2021). The Downstream Effects of Teacher Well-Being Programs: Improvements in Teachers’ Stress, Cognition and Well-Being Benefit Their Students. Front. Psychol..

[159] Nwoko J.C., Emeto T.I., Malau-Aduli A.E.O., Malau-Aduli B.S. (2023). A Systematic Review of the Factors That Influence Teachers’ Occupational Wellbeing. Int. J. Environ. Res. Public Health.

