# Supplementary material for: Food, Quality of Life and Mental Health: A Cross-Sectional Study with Federal Education Workers
Source: Nutrients. 2025 Jul 31;17(15):2519. doi: 10.3390/nu17152519 (PMC12348065; doi:10.3390/nu17152519)
Supplement: Supplementary file 1 [file nutrients-17-02519-s001.zip › nutrients-3691962-supplementary.docx]

Table S1 - Results of the bivariate analysis of the prevalence ratio of regular consumption of ultra-processed foods by civil servants in the Brazilian federal education network (N=1563).

|  | RC Sweets | | | RC Soft drinks | | | RC industrialized/ultra-processed salty foods | | | RC Fast food | | |
| --- | --- | --- | --- | --- | --- | --- | --- | --- | --- | --- | --- | --- |
|  | n (%) | PR (CI 95%) | *p* | n (%) | PR (CI 95%) | *p* | n (%) | PR (CI 95%) | *p* | n (%) | PR (CI 95%) | *p* |
| SOCIODEMOGRAPHIC | | | | | | | | | | | | |
| Sex |  |  |  |  |  |  |  |  |  |  |  |  |
| Male | 138 (20.7) | 1 |  | 59 (8.8) | 1 |  | 103 (15.4) | 1 |  | 14 (2.1) | 1 |  |
| Female | 257 (28.7) | **1,39 (1,16 - 1,67)** | <0.001 | 72 (8) | 0,91 (0,65 - 1,27) | 0,655 | 124 (13.9) | 0,89 (0,70 - 1,14) | 0,385 | 26 (2.9) | 1,38 (0,73 - 2,63) | 0,319 |
| Age group |  |  |  |  |  |  |  |  |  |  |  |  |
| 22-34 | 105 (32.9) | 1 |  | 27 (8.5) | 1 |  | 56 (17.6) | 1 |  | 10 (3.1) | 1 |  |
| 35-47 | 236 (28) | 0,85 (0,70 - 1,03) | 0,098 | 83 (9.9) | 1,16 (0,77 - 1,76) | 0,471 | 130 (15.4) | 0,88 (0,66 - 1,17) | 0,378 | 18 (2.1) | 0,68 (0,32 - 1,46) | 0,325 |
| 48-60 | 47 (13.5) | **0,41 (0,30 - 0,56)** | <0,001 | 15 (4.3) | **0,51 (0,27 - 0,94)** | 0,030 | 34 (9.7) | **0,55 (0,37 - 0,83)** | 0,004 | 12 (3.4) | 1,10 (0,48 - 2,50) | 0,826 |
| 61-72 | 7 (13.2) | **0,40 (0,19 - 0,81)** | 0,011 | 6 (11.3) | 1,34 (0,58 - 3,08) | 0,495 | 7 (13.2) | 0,75 (0,36 - 1,56) | 0,445 | - | - | - |
| Region |  |  |  |  |  |  |  |  |  |  |  |  |
| North-Northeast | 71 (17.3) | 1 |  | 19 (4.6) | 1 |  | 47 (11.4) | 1 |  | 10 (2.4) | 1 |  |
| South | 73 (30.2) | **1,75 (1,31 - 2,32)** | <0,001 | 14 (5.8) | 1,25 (0,64 - 2,45) | 0,513 | 42 (17.4) | **1,52 (1,03 - 2,23)** | 0,034 | 5 (2.1) | 0,85 (0,29 - 2,45) | 0,763 |
| Midwest | 123 (24.4) | **1,41 (1,09 - 1,83)** | 0,010 | 56 (11.1) | **2,40 (1,45 - 3,98)** | 0,001 | 72 (14.3) | 1,25 (0,89 - 1,76) | 0,204 | 17 (3.4) | 1,39 (0,64 - 2,99) | 0,406 |
| Southeast | 128 (31.5) | **1,82 (1,41 - 2,36)** | <0,001 | 42 (10.3) | **2,24 (1,32 - 3,78)** | 0,003 | 66 (16.3) | **1,42 (1,00 - 2,01)** | 0,048 | 8 (2) | 0,81 (0,32 - 2,03) | 0,653 |
| Marital status |  |  |  |  |  |  |  |  |  |  |  |  |
| Married | 242 (23.7) | 1 |  | 90 (8.8) | 1 |  | 145 (14.2) | 1 |  | 21 (2.1) | 1 |  |
| Single | 114 (30.2) | **1,27 (1,05 - 1,54)** | 0,012 | 28 (7.4) | 0,84 (0,56 - 1,26) | 0,404 | 57 (15.1) | 1,06 (0,80 - 1,41) | 0,674 | 15 (4) | **1,93 (1,01 - 3,31)** | 0,048 |
| Divorced/Widoed | 39 (23.4) | 0,98 (0,73 - 1,32) | 0,911 | 13 (7.8) | 0,88 (0,50 - 1,54) | 0,657 | 25 (15) | 1,05 (0,71 - 1,56) | 0,800 | 4 (2.4) | 1,16 (0,40 - 3,34) | 0,780 |
| WORK AND TRAINING | | | | | | | | | | | | |
| Level of education |  |  |  |  |  |  |  |  |  |  |  |  |
| Doctorate/PHD | 168 (26.4) | 1 |  | 56 (8.8) | 1 |  | 91 (14.3) | 1 |  | 15 (2.4) | 1 |  |
| Master’s degree | 28 (25.2) | 0,95 (0,68 - 1,35) | 0,068 | 9 (8.1) | 0,92 (0,47 - 1,80) | 0,811 | 23 (20.7) | 1,45 (0,96 - 2,18) | 0,077 | 4 (3.6) | 1,53 (0,52 - 4,52) | 0,444 |
| Specialization /MBA | 122 (25.2) | 0,95 (0,78 - 1,17) | 0,209 | 42 (8.7) | 0,99 (0,67 - 1,44) | 0,940 | 71 (14.7) | 1,02 (0,77 - 1,37) | 0,865 | 11 (2.3) | 0,96 (0,45 - 2,08) | 0,925 |
| HS/HE/PTE | 77 (23.2) | 0,88 (0,70 - 1,11) | 1,179 | 24 (7.2) | 0,82 (0,52 - 1,30) | 0,400 | 42 (12.7) | 0,88 (0,63 - 1,24) | 0,479 | 10 (3) | 1,28 (0,58 - 2,81) | 0,543 |
| Position |  |  |  |  |  |  |  |  |  |  |  |  |
| ATE | 215 (24.5) | 1 |  | 71 (8.1) | 1 |  | 125 (14.2) | 1 |  | 20 (2.3) | 1 |  |
| Teachers | 180 (26.3) | 1,07 (0,90 - 1,27) | 0,419 | 60 (8.8) | 1,08 (0,78 - 1,50) | 0,634 | 102 (14.9) | 1,05 (0,82 - 1,33) | 0,716 | 20 (2.9) | 1,28 (0,69 - 2,36) | 0,426 |
| Length of service |  |  |  |  |  |  |  |  |  |  |  |  |
| ≥11 years | 119 (20.7) | 1 |  | 44 (7.6) | 1 |  | 71 (12.3) | 1 |  | 18 (3.1) | 1 |  |
| 6 - 10 years | 177 (26.3) | **1,27 (1,04 - 1,56)** | 0,021 | 66 (9.8) | 1,28 (0,89 - 1,85) | 0,182 | 108 (16) | 1,30 (0,98 - 1,72) | 0,064 | 13 (1.9) | 0,62 (0,30 - 1,25) | 0,180 |
| 1 - 5 years | 99 (31.6) | **1,53 (1,22 - 1,92)** | <0,001 | 21 (6.7) | 0,88 (0,53 - 1,45) | 0,612 | 48 (15.3) | 1,24 (0,89 - 1,75) | 0,207 | 9 (2.9) | 0,92 (0,42 - 2,02) | 0,836 |
| BODY PERCEPTION AND LIFESTYLE HABITS | | | | | | | | | | | | |
| Body satisfaction |  |  |  |  |  |  |  |  |  |  |  |  |
| 1° tercil - satisfied | 181 (22.0) | 1 |  | 56 (6.8) | 1 |  | 92 (11.2) | 1 |  | 26 (3.2) | 1 |  |
| 2° tercil - neutral | 122 (25.8) | 1,17 (0,96 - 1,43) | 0,113 | 39 (8.3) | 1,21 (0,82 - 1,80) | 0,333 | 71 (15) | **1,35 (1,01 - 1,79)** | 0,043 | 6 (1.3) | **0,40 (0,17 - 0,97)** | 0,043 |
| 3° tercil - dissatisfied | 92 (34.3) | **1,56 (1,27 - 1,92)** | <0,001 | 36 (13.4) | **1,97 (1,33 - 2,93)** | 0,001 | 64 (32.9) | **2,14 (1,60 - 2,85)** | <0,001 | 8 (3.0) | 0,94 (0,43 - 2,06) | 0,887 |
| Quality of sleep |  |  |  |  |  |  |  |  |  |  |  |  |
| 1° tercil - satisfied | 172 (23.3) | 1 |  | 52 (7) | 1 |  | 90 (12.2) | 1 |  | 16 (2.2) | 1 |  |
| 2° tercil - neutral | 92 (22.8) | 0,98 (0,78 - 1,22) | 0,855 | 33 (8.2) | 1,16 (0,76 - 1,78) | 0,482 | 50 (12.4) | 1,02 (0,74 - 1,41) | 0,917 | 8 (2) | 0,92 (0,39 - 2,12) | 0,837 |
| 3° tercil - dissatisfied | 131 (31) | **1,33 (1,09 - 1,61)** | 0,004 | 46 (10.9) | **1,55 (1,06 - 2,26)** | 0,024 | 87 (20.6) | **1,70 (1,29 - 2,21)** | <0,001 | 16 (3.8) | 1,75 (0,88 - 3,46) | 0,108 |
| Hours of sleep |  |  |  |  |  |  |  |  |  |  |  |  |
| 7 - 8h | 204 (25) | 1 |  | 64 (7.9) | 1 |  | 103 (12.6) | 1 |  | 17 (2.1) | 1 |  |
| ≥9h | 19 (31.1) | 1,24 (0,84 - 1,84) | 0,274 | 8 (13.1) | 1,67 (0,84 - 3,32) | 0,144 | 8 (13.1) | 1,04 (0,53 - 2,03) | 0,914 | - | - | - |
| ≤6h | 172 (25) | 1,00 (0,84 - 1,19) | 0,998 | 59 (8.6) | 1,09 (0,78 - 1,53) | 0,605 | 116 (16.9) | **1,34 (1,05 - 1,71)** | 0,021 | 23 (3.3) | 1,60 (0,86 - 2,98) | 0,134 |
| TV time (h/d) |  |  |  |  |  |  |  |  |  |  |  |  |
| <1h | 144 (26.6) | 1 |  | 55 (10.2) | 1 |  | 72 (13.3) | 1 |  | 14 (2.6) | 1 |  |
| 1 - 2h | 148 (24.7) | 0,93 (0,76 - 1,13) | 0,471 | 56 (9.4) | 0,92 (0,65 - 1,31) | 0,649 | 85 (14.2) | 1,07 (0,80 - 1,43) | 0,658 | 15 (2.5) | 0,97 (0,47 - 1,99) | 0,932 |
| ≥3h | 85 (24.9) | 0,93 (0,74 - 1,18) | 0,561 | 14 (4.1) | **0,40 (0,23 - 0,71)** | 0,002 | 57 (16.7) | 1,25 (0,91 - 1,72) | 0,168 | 8 (2.3) | 0,90 (0,38 - 2,13) | 0,818 |
| Regular physical activity |  |  |  |  |  |  |  |  |  |  |  |  |
| Yes | 187 (21.5) | 1 |  | 43 (4.9) | 1 |  | 96 (11) | 1 |  | 13 (1.5) | 1 |  |
| No | 208 (30) | **1,33 (1,12 - 1,59)** | <0,001 | **88 (12.7)** | **2,56 (1,80 - 3,64)** | <0,001 | **131 (18.9)** | **1,71 (1,34 - 2,18)** | <0,001 | **27 (3.9)** | **2,60 (1,35 - 5,00)** | 0,004 |
| Weekly frequency of physical activity |  |  |  |  |  |  |  |  |  |  |  |  |
| ≥4 days | 63 (17.3) | 1 |  | 14 (3.8) | 1 |  | 34 (9.3) | 1 |  | 6 (1.6) | 1 |  |
| 1 - 3 days | 120 (24.7) | **1,43 (1,09 - 1,88)** | 0,010 | 28 (5.8) | 1,50 (0,80 - 2,82) | 0,201 | 60 (12.4) | 1,33 (0,89 - 1,98) | 0,163 | 7 (1.4) | 0,88 (0,30 - 2,59) | 0,814 |
| 0 day | 208 (30) | **1,74 (1,35 - 2,23)** | <0,001 | 88 (12.7) | **3,31 (1,91 - 5,73)** | <0,001 | 131 (18.9) | **2,03 (1,42 - 2,89)** | <0,001 | 27 (3.9) | 2,37 (0,99 - 5,68) | 0,054 |
| MENTAL HEALTH - DASS-21 | | | | | | | | | | | | |
| Stress |  |  |  |  |  |  |  |  |  |  |  |  |
| Normal | 187 (21.8) | 1 |  | 51 (6) | 1 |  | 94 (11) | 1 |  | 17 (2) | 1 |  |
| Moderate | 116 (28.6) | **1,31 (1,07 - 1,60)** | 0,008 | 40 (9.9) | **1,65 (1,11 - 2,46)** | 0,013 | 62 (15.3) | **1,39 (1,03 - 1,87)** | <0,001 | 13 (3.2) | 1,61 (0,79 - 3,29) | 0,189 |
| High | 92 (30.6) | **1,40 (1,13 - 1,73)** | 0,002 | 40 (13.3) | **2,23 (1,51 - 3,30)** | <0,001 | 71 (14.5) | **2,15 (1,62 - 2,84)** | 0,030 | 10 (3.3) | 1,67 (0,77 - 3,61) | 0,190 |
| Anxiety |  |  |  |  |  |  |  |  |  |  |  |  |
| Normal | 194 (22.4) | 1 |  | 55 (6.4) | 1 |  | 100 (11.5) | 1 |  | 17 (2) | 1 |  |
| Moderate | 100 (25.3) | **1,24 (1,01 - 1,52)** | 0,045 | 37 (10.2) | **1,61 (1,08 - 2,40)** | 0,019 | 56 (15.5) | 1,34 (0,99 - 1,82) | 0,056 | 11 (27.5) | 1,55 (0,73 - 3,28) | 0,250 |
| High | 101 (30.1) | **1,34 (1,09 - 1,65)** | 0,005 | 39 (11.6) | **1,83 (1,24 - 2,70)** | 0,002 | 71 (21.1) | **1,83 (1,39 - 2,41)** | <0,001 | 12 (3.6) | 1,82 (0,88 - 3,77) | 0,107 |
| Depression |  |  |  |  |  |  |  |  |  |  |  |  |
| Normal | 175 (21.1) | 1 |  | 53 (6.4) | 1 |  | 83 (10) | 1 |  | 19 (2.3) | 1 |  |
| Moderate | 139 (30) | **1,42 (1,17 - 1,72)** | <0,001 | 40 (8.6) | 1,35 (0,91 - 2,00) | 0,134 | 75 (16.2) | **1,62 (1,21 - 2,16)** | 0,001 | 11 (2.4) | 1,04 (0,50 - 2,16) | 0,923 |
| High | 81 (30.2) | **1,43 (1,15 - 1,80)** | 0,002 | 38 (14.2) | **2,22 (1,50 - 3,29)** | <0,001 | 69 (25.7) | **2,58 (1,93 - 3,44)** | <0,001 | 10 (3.7) | 1,63 (0,77 - 3,47) | 0,203 |
| QUALITY OF LIFE - Whoquol-breaf | | | | | | | | | | | | |
| Physics |  |  |  |  |  |  |  |  |  |  |  |  |
| 3rd tertile - better | 101 (22) | 1 |  | 33 (7.2) | 1 |  | 54 (11.7) | 1 |  | 9 (2) | 1 |  |
| 2nd tertile | 132 (24.5) | 1,11 (0,89 - 1,40) | 0,346 | 33 (6.1) | 0,85 (0,53 - 1,36) | 0,505 | 70 (13) | 1,11 (0,79 - 1,54) | 0,551 | 14 (2.6) | 1,33 (0,58 - 3,04) | 0,502 |
| 1st tertile - worse | 162 (28.7) | **1,31 (1,05 - 1,62)** | 0,015 | 65 (11.5) | **1,65 (1,07 - 2,40)** | 0,020 | 103 (18.3) | **1,56 (1,14 - 2,11)** | 0,005 | 17 (3) | 1,54 (0,69 - 3,42) | 0,289 |
| Psychological |  |  |  |  |  |  |  |  |  |  |  |  |
| 3rd tertile - better | 126 (19.5) | 1 |  | 40 (6.2) | 1 |  | 61 (9.4) | 1 |  | 16 (2.5) | 1 |  |
| 2nd tertile | 119 (26.7) | **1,37 (1,10 - 1,71)** | 0,005 | 33 (7.4) | 1,20 (0,77 - 1,87) | 0,432 | 58 (13) | 1,38 (0,98 - 1,94) | 0,061 | 13 (2.9) | 1,18 (0,57 - 2,43) | 0,651 |
| 1st tertile - worse | 150 (31.8) | **1,63 (1,33 - 2,01)** | <0,001 | 58 (12.3) | **1,99 (1,35 - 2,93)** | <0,001 | 108 (22.9) | **2,43 (1,82 - 3,25)** | <0,001 | 11 (2.3) | 0,94 (0,44 - 2,02) | 0,882 |
| Social |  |  |  |  |  |  |  |  |  |  |  |  |
| 3rd tertile - better | 69 (24.4) | 1 |  | 24 (8.5) | 1 |  | 34 (12) | 1 |  | 7 (2.5) | 1 |  |
| 2nd tertile | 196 (26.8) | 1,10 (0,87 - 1,39) | 0,433 | 46 (6.3) | 0,74 (0,46 - 1,19) | 0,217 | 91 (12.4) | 1,04 (0,72 - 1,50) | 0,850 | 19 (2.6) | 1,05 (0,45 - 2,47) | 0,910 |
| 1st tertile - worse | 130 (23.7) | 0,97 (0,75 - 1,25) | 0,822 | 61 (11.1) | 1,31 (0,83 - 2,05) | 0,239 | 102 (18.6) | **1,55 (1,08 - 2,22)** | 0,018 | 14 (2.6) | 1,03 (0,42 - 2,52) | 0,947 |
| Environmental |  |  |  |  |  |  |  |  |  |  |  |  |
| 3rd tertile - better | 112 (25.6) | 1 |  | 38 (8.7) | 1 |  | 53 (12.1) | 1 |  | 11 (2.5) | 1 |  |
| 2nd tertile | 157 (26.2) | 1,02 (0,83 - 1,26) | 0,833 | 48 (8) | 0,92 (0,61 - 1,38) | 0,694 | 76 (12.7) | 1,05 (0,75 - 1,45) | 0,788 | 13 (2.2) | 0,86 (0,39 - 1,91) | 0,714 |
| 1st tertile - worse | 126 (23.9) | 0,93 (0,75 - 1,16) | 0,537 | 45 (8.5) | 0,98 (0,65 - 1,48) | 0,931 | 98 (18.6) | **1,53 (1,12 - 2,09)** | 0,007 | 16 (3) | 1,21 (0,57 - 2,57) | 0,628 |
| Total score |  |  |  |  |  |  |  |  |  |  |  |  |
| 3rd tertile - better | 113 (21.6) | 1 |  | 32 (6.1) | 1 |  | 55 (10.5) | 1 |  | 13 (2.5) | 1 |  |
| 2nd tertile | 139 (26.8) | 1,24 (0,99 - 1,54) | 0,052 | 41 (7.9) | 1,29 (0,83 - 2,02) | 0,262 | 62 (11.9) | 1,14 (0,81 - 1,60) | 0,465 | 11 (2.1) | 0,85 (0,39 - 1,89) | 0,694 |
| 1st tertile - worse | 143 (27.4) | **1,27 (1,02 - 1,57)** | 0,029 | 58 (11.1) | **1,82 (1,20 - 2,75)** | 0,005 | 110 (21.1) | **2,01 (1,49 - 2,71)** | <0,001 | 16 (3.1) | 1,23 (0,60 - 2,54) | 0,566 |

Note: The “n” column represents absolute frequencies, while the “%” column represents relative frequencies. “N” represents the sample size. The analysis was conducted using the Poisson regression model with robust variance. The measure of effect is the PR with its respective 95% CI. Higher scores on the WHOQOL-bref indicated better quality of life. “HS/HE/PTE”: High School/Higher Education/Professional and Technological Educationand ‘MBA’ for Master's in Business Administration.
